# Systemic delivery of AAVrh74.tMCK.hCAPN3 rescues the phenotype in a mouse model for LGMD2A/R1

**DOI:** 10.1016/j.omtm.2021.06.010

**Published:** 2021-06-24

**Authors:** Zarife Sahenk, Burcak Ozes, Darren Murrey, Morgan Myers, Kyle Moss, Mehmet E. Yalvac, Alicia Ridgley, Lei Chen, Jerry R. Mendell

**Affiliations:** 1 Center for Gene Therapy, The Abigail Wexner Research Institute, Nationwide Children’s Hospital, 700 Children’s Drive, Rm. WA 3024, Columbus, OH 43205, USA; 2 Department of Pediatrics and Neurology, Nationwide Children’s Hospital and The Ohio State University, Columbus, OH 43205, USA; 3 Department of Pathology and Laboratory Medicine, Nationwide Children’s Hospital, Columbus, OH 43205, USA; 4 Department of Neurology, The Ohio State University, Wexner Medical Center, Columbus, OH 43210, USA

**Keywords:** LGMD2A/R1, calpainopathy, calpain3, AAV, muscular dystrophy, gene therapy, functional correlation

## Abstract

Limb girdle muscular dystrophy (LGMD) 2A/R1, caused by mutations in the *CAPN3* gene and CAPN3 loss of function, is known to play a role in disease pathogenicity. In this study, AAVrh74.tMCK.CAPN3 was delivered systemically to two different age groups of CAPN3 knockout (KO) mice; each group included two treatment cohorts receiving low (1.17 × 10^14^ vg/kg) and high (2.35 × 10^14^ vg/kg) doses of the vector and untreated controls. Treatment efficacy was tested 20 weeks after gene delivery using functional (treadmill), physiological (*in vivo* muscle contractility assay), and histopathological outcomes. AAV.CAPN3 gene therapy resulted in significant, robust improvements in functional outcomes and muscle physiology at low and high doses in both age groups. Histological analyses of skeletal muscle showed remodeling of muscle, a switch to fatigue-resistant oxidative fibers in females, and fiber size increases in both sexes. Safety studies revealed no organ tissue abnormalities; specifically, there was no histopathological evidence of cardiotoxicity. These results show that *CAPN3* gene replacement therapy improved the phenotype in the CAPN3 KO mouse model at both doses independent of age at the time of vector administration. The improvements were supported by an absence of cardiotoxicity, showing the efficacy and safety of the AAV.CAPN3 vector as a potential gene therapy for LGMDR1.

## Graphical abstract

**Figure:**
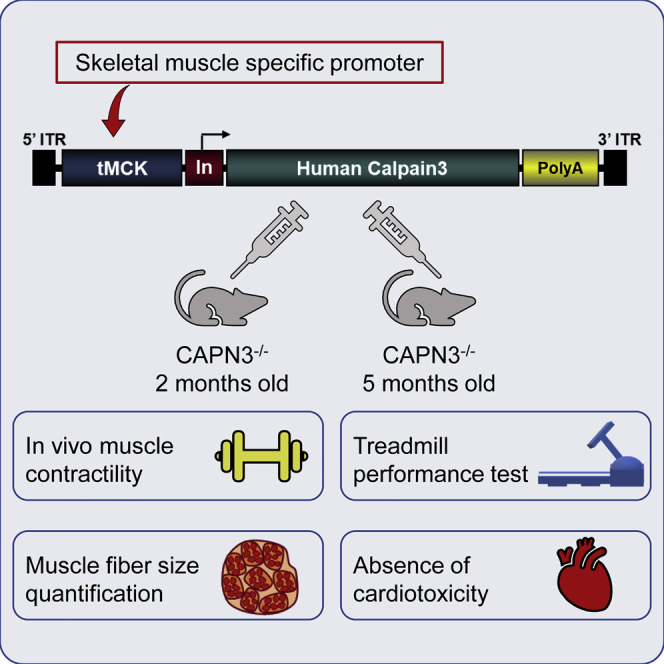

## Teaser

The AAVrh74.tMCK.CAPN3 vector was delivered to two different age groups of the LGMD2A/R1 mouse model, CAPN3 KO mice. 20 weeks after injection, therapy resulted in improvements in functional (treadmill), physiological (*in vivo* muscle contractility), and histopathological outcomes without cardiotoxicity, showing the efficacy and safety of the AAV.CAPN3 vector as a potential gene therapy for LGMDR1.

## Introduction

Limb-girdle muscular dystrophy recessive 1 (LGMDR1), previously known as LGMD2A (calpainopathy), is the most common LGMD subtype worldwide and is caused by mutations in the *CAPN3* gene, which encodes a skeletal muscle-specific, Ca^2+^-activated, nonlysosomal cysteine protease, calpain 3 (CAPN3).^1^ The current estimated prevalence of LGMD2A/R1 is 8.4 (6.8–10.2) per million, based on disease prevalence known to capture pathogenic and loss-of-function variants using public sequencing databases.^2^ LGMDR1 is characterized by progressive symmetric weakness of the shoulder, pelvic, and proximal limb muscles without cardiac or pulmonary manifestations.^3^ The onset of weakness is variable but usually begins in children and young adults and results in loss of ambulation within 20 years after disease onset in most individuals. The pathophysiological mechanisms involved in LGMDR1 remain unclear. The full length of CAPN3 is expressed specifically in skeletal muscle,^4^ and the enzyme, present in its inactive form, is capable of self-activation by autolysis, which exposes the catalytic site for substrate accessibility.^5^^,^^6^ CAPN3 is involved in cleavage and/or breakdown of multiple key skeletal muscle proteins, particularly those involved in assembly and scaffolding of myofibrillar organization, such as titin, vinculin, C-protein, and others.^5^^,^^7^^,^^8^ Loss of this activity, which is presumably involved in sarcomere maintenance and turnover, has been implicated in the pathogenesis of LGMDR1.^7^^,^^9^, ^10^, ^11^ In addition, CAPN3 possesses thiol-dependent proteolytic activity directed specifically against the skeletal muscle ryanodine receptor 1 (RyR1), a Ca^2+^ release channel localized at the sarcoplasmic reticulum (SR) terminal cisternae.^12^^,^^13^ It has been proposed that the dysregulation of skeletal muscle functions in LGMDR1 is, at least in part, a consequence of the lack of RyR stabilization by CAPN3.^13^, ^14^, ^15^, ^16^ The absence of functional CAPN3 has been shown to result in reduced levels of several important Ca^2+^-handling proteins, such as RyR1 and SR/endoplasmic reticulum Ca^2+^-ATPase, SERCA^17^, and impaired Ca^2+^/calmodulin-dependent protein kinase type II (CaMKII) signaling, therefore affecting Ca^2+^-dependent transcriptional pathways that control muscle growth, fiber transition, or mitochondrial biogenesis.^18^, ^19^, ^20^, ^21^ Accumulating evidence suggests that dysregulation of Ca^2+^ homeostasis in skeletal muscle may be a significant underlying event in LGMDR1,^22^ leading to mitochondrial abnormalities/dysfunction,^18^^,^^21^^,^^23^^,^^24^ increased oxidative stress,^23^^,^^25^ reduced energy production, and impaired muscle regeneration and adaptation.^19^^,^^21^^,^^26^

At present, there is no treatment for LGMDR1. Evidence indicates that this disorder is caused by CAPN3 loss of function; therefore, *CAPN3* gene transfer for LGMDR1 is a feasible therapeutic strategy. Previous studies have demonstrated the potential of *CAPN3* gene delivery to correct the pathological signs following intramuscular (IM) delivery in a *Capn3*-deficient mouse model.^27^ Earlier studies using systemic gene delivery, designed for expression of *CAPN3* driven by the desmin promoter in wild-type (WT) mice resulted in cardiotoxicity; however, this effect was circumvented by introducing a target sequence of the heart-specific microRNA-208a in the cassette, suppressing *CAPN3* transgene expression in the cardiac tissue.^28^ Using a cardiotoxin-induced regeneration paradigm in *Capn3*-null muscle, we previously demonstrated that the absence of CAPN3 is associated with impaired muscle regeneration, which is an important pathological feature of LGMDR1, and that IM delivery of a *CAPN3* gene construct reversed this defect by regulating radial growth of regenerating fibers.^21^ In the current study, we used a triple muscle-specific creatine kinase (tMCK) promoter^29^ to largely restrict *CAPN3* expression to skeletal muscle and a single-stranded adeno-associated virus serotype rh74 (ssAAVrh74) as the delivery vector, validated as suitable for systemic administration of *CAPN3.* We tested the efficacy of this systemically delivered vector in *Capn3*-null mice (CAPN3 knockout [KO]) using functional, physiological, and histopathological outcomes. These results show a long-term therapeutic benefit of AAVrh74.tMCK.hCAPN3 in CAPN3 KO mice following systemic delivery at low and high doses in young and old age groups, along with toxicology and biodistribution studies providing proof-of-principle preclinical data for potential CAPN3 gene therapy for LGMDR1.

## Results

### rAAVrh74.tMCK.hCAPN3 vector production, potency, and design of experimental cohorts

**Figure 1:**
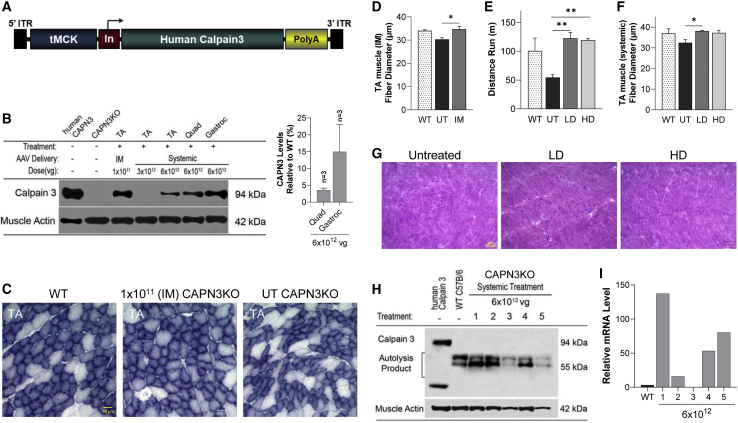
Biopotency of the AAVrh74.tMCK.CAPN3 vector, short-term efficacy, and cardiac safety 4 weeks after gene delivery (A) Schematic of the AAVrh74.tMCK.CAPN3 vector showing 5′ and 3′ AAV2 inverted terminal repeats (ITRs), the tMCK promoter, the chimeric intron (In), the human CAPN3 transgene, and the SV40 polyadenylation signal (poly(A)). (B) Representative western blot images of CAPN3 protein in muscle samples following intramuscular (IM) and systemic gene delivery in CAPN3 KO mice with normal human muscle lysate (gel load of 60% total protein compared with mouse lysates from *tibialis anterior* [TA], quadriceps, and gastrocnemius), and UT CAPN3 KO mice for comparison. 94-kDa, full-length human CAPN3 was detected in the TA 4 weeks after IM injection of the vector at 1 × 10^11^ vg. Following systemic delivery, note the prominent increase of full-length CAPN3 protein from the 6 × 10^12^ vg treatment (high dose [HD]) compared with the 3 × 10^12^ vg (low dose [LD]) cohort. 12.5 μg and 2.5 μg (25% and 5%, respectively) total protein from the corresponding UT WT C57BL/6 muscles were used as reference points for semiquantitative analysis of the AAV-mediated CAPN3 levels in the corresponding treated CAPN3 KO mouse muscles. CAPN3 levels were detected using the CALP-12A2 antibody, which recognizes epitopes in muscle extracts from human and mouse. (C and D) CAPN3 protein levels in the quadriceps (C) and gastrocnemius (D) are quantified as percent of the WT, and the levels of all HD samples (n = 3) are given as mean ± SEM. (C) Representative images of succinic dehydrogenase (SDH)-stained tissue sections from TA muscle in wild-type (WT) and CAPN3 KO mice following IM delivery of AAV.hCAPN3 or Ringer’s lactate (untreated [UT]). (D) A fiber size increase toward normalization was seen with CAPN3 gene therapy. (E) Following systemic AAV.hCAPN3 gene therapy, LD- and HD-treated CAPN3 KO mice performed better on the treadmill test compared with UT counterparts. (F) The mean fiber diameter of TA muscle was increased toward WT values following systemic delivery in LD and HD cohorts. (G) Representative images from H&E-stained fresh-frozen sections through left ventricles inCAPN3 KO mice 4 weeks after systemic injection of the vector at LD and HD with UT control. Necrosis, regeneration, or inflammation was not seen. (H) Western blot analysis of cardiac tissue from the HD cohort showed no detectable CAPN3 protein. A full-length, 94-kDa hCAPN3 band is present in human skeletal muscle as a positive control. (I) Bar graph representing cardiac mRNA levels of individual mice corresponding to samples shown in western blot. Each bar represents the mean ± SEM of 3–4 mice. ∗∗p < 0.01, one-way ANOVA, Tukey’s multiple comparisons test. Scale bars 30 μm in the WT image (applies to all).

The vector used in these studies was produced at the Abigail Wexner Research Institute at Nationwide Children’s Hospital. Studies were carried out using the AAVrh74 vector carrying the human *CAPN3* cDNA coding sequence consisting of 24 exons under control of the tMCK promoter. Figure 1A shows the cassette used for gene transfer, including a tMCK promoter, a chimeric intron to enhance gene expression, the full human *CAPN3* cDNA, and the SV40 polyadenylation signal.

*In vivo* biopotency testing was carried out first, using the IM route following injection of AAVrh74.tMCK.hCAPN3 (1 × 10^11^ vg) into the *tibialis anterior* muscle in CAPN3 KO mice (n = 3) 4 weeks after gene delivery. qRT-PCR and western blot analyses showed human *CAPN3* transcripts and 94-kDa full-length total levels of CAPN3 protein (Figure 1B), indicating that production of CAPN3 was achieved. Moreover, histological analysis showed that CAPN3 replacement increased the muscle fiber diameter of the *tibialis anterior* compared with the control (Ringer’s lactate injected) muscle (Figures 1C and 1D), confirming *in vivo* CAPN3 functionality.

As the next step, we assessed the biopotency of the vector following systemic delivery via the tail vein of CAPN3 KO mice at low and high doses (low dose [LD], 3 × 10^12^ vg, n = 4; high dose [HD], 6 × 10^12^ vg, n = 3) and generated biodistribution and short-term treatment efficacy data 4 weeks after injection for both cohorts. *hCAPN3* expression in CAPN3 KO muscle following systemic delivery was variable among different muscles and especially in the LD cohort, which was significantly lower compared with IM delivery at 1 × 10^11^ vg (<1% of IM delivery for *tibialis anterior* muscle). Accordingly, the full-length, 94-kDa protein was below the limit of detection by western blot (Figure 1B). However, robust gene expression and prominent amounts of full-length CAPN3 protein were exhibited following the 6 × 10^12^ vg systemic dosage in the HD cohort, suggesting a dose-dependent response following AAVrh74.tMCK.hCAPN3 delivery (Figure 1B). Although the number of CAPN3 KO mice used in the biopotency experiments was small, additional data from these mice were obtained to assess whether short-term functional or histological efficacy could be detected. We observed improvements in these parameters in both cohorts of CAPN3 KO mice that received 3 × 10^12^ or 6 × 10^12^ vg of vector systemically 4 weeks after gene injection compared with Ringer’s lactate-injected (n = 3) counterparts (Figures 1E and 1F). In the run-to-exhaustion test, both treatment cohorts improved significantly compared with the untreated cohort, and there was no dose-related difference in performance. Accompanying this, muscle fiber diameter showed an increase toward normalization in both cohorts, again with no dose-related differences (Table S1). In addition, we found no histopathological evidence of cardiac toxicity following systemic injection of the AAVrh7.4.tMCK.hCAPN3 vector at 4 weeks in either cohort. Microscopy examination of sections from two levels (superficial and deep sections from ventricles) through the apex of the heart revealed no inflammation, necrosis, regeneration, or fibrotic changes in the cardiac muscle, indicating no short-term cardiotoxic effects from systemic delivery of the vector at these two doses (Figure 1G). We found no detectable 94-kDa CAPN3 protein bands in the heart tissue by western blot in either cohort (Figure 1H) and highly variable mRNA levels between animals (Figure 1I). These observations guided us to test the long-term efficacy of systemic AAVrh74.tMCK.hCAPN3 gene therapy at the same LD and HD in young and old age groups of CAPN3 KO mice using functional and *in vivo* muscle physiology and histological outcomes.

### Long-term treatment efficacy of rAAVrh74.tMCK.hCAPN3 following systemic injection at LD and HD

AAVrh74.tMCK.CAPN3 was systemically delivered to two different age groups of CAPN3 KO mice, 6–10 weeks old and 20–24 weeks old. Each group included two treatment cohorts receiving an LD (3 × 10^12^ vg) and HD (6 × 10^12^ vg) of the vector and age-matched untreated (UT) control CAPN3 KO mice. Treatment efficacy was tested 20 weeks after gene delivery using functional (treadmill, run-to-exhaustion test), physiological (*in vivo* muscle contractility assay), and histopathological outcomes. Table S2 summarizes the experimental cohorts in each age group.

#### Run-to-exhaustion test

**Figure 2:**
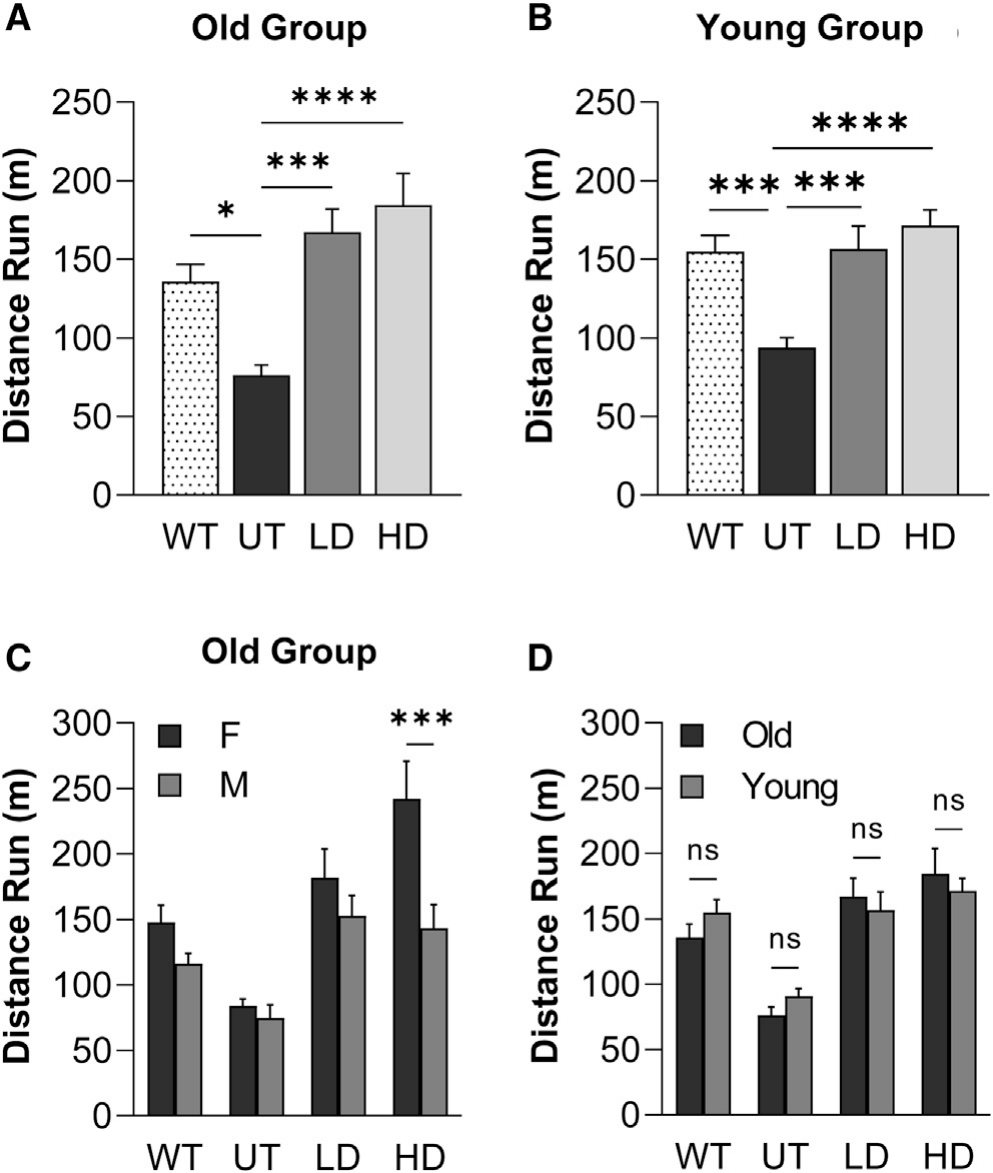
Functional efficacy of AAV.CAPN3 gene therapy by treadmill performance (A and B) In the treadmill test, the LD (3 – 10^12^ vg) and HD (6 × 10^12^ vg) treatment cohorts showed significant improvements in (A) old and (B) young age groups compared with UT CAPN3 KO mice, reaching performance levels of age-matched WT mice. (C) Females of the HD cohort from the old group performed significantly better than the males of the same group. (D) Age at onset of treatment did not show a significant difference in treadmill outcomes. Each bar represents the mean ± SEM of 12–14 mice per CAPN3 KO cohort and 8–14 mice per age-matched WT mouse. ∗∗p < 0.01, ∗∗∗p < 0.001, ∗∗∗∗p < 0.0001, one-way ANOVA, Tukey’s multiple comparisons test for (A) and (B) and two-way ANOVA, Sidak’s multiple comparison test for (C) and (D).

The run-to-exhaustion treadmill test was used to assess the functional efficacy of CAPN3 gene therapy. At both doses in young and older age groups, gene therapy improved treadmill performance 20 weeks after gene delivery, leading to striking increases in run distance comparable with age-matched WT performance compared with the UT CAPN3 KO cohort (Figure 2A and B). In the older age group, CAPN3 gene therapy led to a 119% increase in distance run to exhaustion in the LD cohort (167.2 ± 14.1 m, n = 12) and a 142% increase in the HD cohort (184.5 ± 19.2 m, n = 12) compared with the UT cohort (76.2 ± 6.2 m, n = 14; p = 0.0001 for UT versus LD and p < 0.0001 for UT versus HD; 135.9 ± 10.3 m, n = 8 for WT, p = 0.035 for WT versus UT; Figure 2A). Similarly, in the young age group, treadmill data indicated a 72% performance increase in the LD cohort (156.7 ± 14.0 m, n = 12) and an 88% increase in the HD cohort (171.4 ± 9.6 m, n = 12) compared with the UT cohort (93.9 ± 6.0 m, n = 12; p = 0.0009 for UT versus LD and p < 0.0001 for UT versus HD; 155.0 ± 9.7 m, n = 13 for WT, p = 0.0010 for WT versus UT; Figure 2B). We observed that the UT cohort of the young age group performed better than the UT counterparts from the old age group without reaching statistical significance. Treadmill performance did not differ significantly among the treated cohorts, suggesting that the AAVrh74.tMCK.CAPN3 vector at the LD was sufficient to give rise to functional improvements comparable with the HD cohorts. On the other hand, comparing male (n = 7) and female (n = 5) performance at the HD, the females performed significantly better (p = 0.0006; Figure 2C), whereas in the young group, gender difference did not influence treadmill performance. There was no significant difference in treadmill outcomes resulting from early-onset treatment (Figure 2D). These results indicate that age at the time of vector administration and the vector dose did not affect the treadmill performance in this model.

#### *In vivo* muscle contractility assay

An *in vivo* muscle contractility assay was used to assess muscle physiology in response to CAPN3 gene therapy. This assay measures the aggregate torque produced by stimulating the tibial nerve to achieve gastrocnemius muscle torque around the ankle joint.

**Figure 3:**
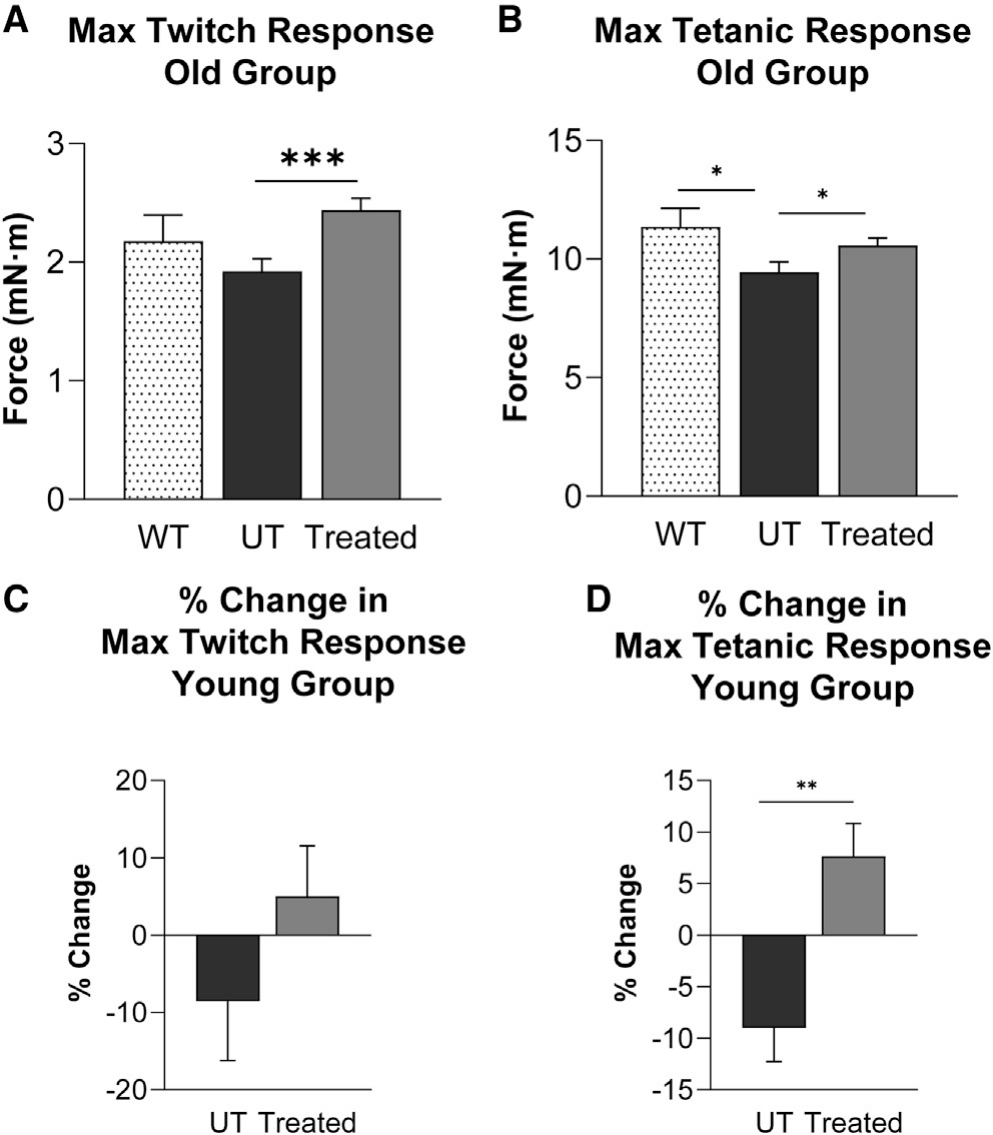
Improvements in muscle physiology with AAV.CAPN3 gene therapy (A and B) *In vivo* muscle contractility assay showing a higher force output in (A) maximum twitch and (B) tetanic responses from gastrocnemius muscle in LD and HD treatment cohorts compared with UT CAPN3 KO mice from the old age group. Data reflect all treatment groups combined (treated cohorts, n = 23; UT cohort, n = 28; WT, n = 10). (C and D) Similarly, treated mice showed increased (C) twitch and (D) tetanic responses in the young age group (treated cohorts, n = 22–23; UT cohort, n = 13). Each bar represents the mean ± SEM. ∗p ≤ 0.05, ∗∗p ≤ 0.01, ∗∗∗p ≤ 0.001, unpaired t test.

In the old group of CAPN3 KO mice, varying degrees of heel cord contractures were observed in the UT males as part of the aged CAPN3 KO phenotype, recapitulating phenotypical differences between males and females in humans.^30^ The contractures were also observed in males of the LD treatment cohort at the endpoint (4 of 6 mice); however, males in the HD cohort were rescued with gene therapy. Taking this variability into consideration, we compared the HD and LD combination treatment cohort (n = 23) with the UT cohort (n = 28) for analysis of *in vivo* muscle contractility data. Treated mice displayed a higher force for maximum twitch and tetanic responses reaching WT levels (2.18 ± 0.2 mN⋅m for twitch, 11.36 ± 0.7 mN⋅m for tetanic, n = 10). The maximum twitch response of the treated group (2.43 ± 0.1 mN⋅m) showed a 27% increase (p = 0.00101), and maximum tetanic response (10.58 ± 0.3 mN⋅m) improved by 12% (p = 0.047) compared with the UT group (twitch, 1.92 ± 0.1 mN⋅m; tetanic, 9.44 ± 0.4 mN⋅m; Figures 3A and 3B).

In the young group of CAPN3 KO mice, which displayed a milder phenotype to begin with (see treadmill performance of UT cohorts from young versus old age groups in Figures 2A and 2B), we assessed the time-course change of muscle contractility by comparing baseline measurements with the endpoint 20 weeks after treatment. The percent changes of twitch and tetanic responses were calculated for each mouse. With treatment, the average percent increase in maximum twitch response was 5% in the combination treatment group (n = 23), whereas an average 9% decrease occurred in the UT group (n = 13, p = 0.20; Figure 3C). We observed an 8% increase in the average percent maximum tetanic response in treated mice (n = 22), whereas the UT group (n = 13) had a 9% decrease (p = 0.002; Figure 3D).

#### Histopathology

**Figure 4:**
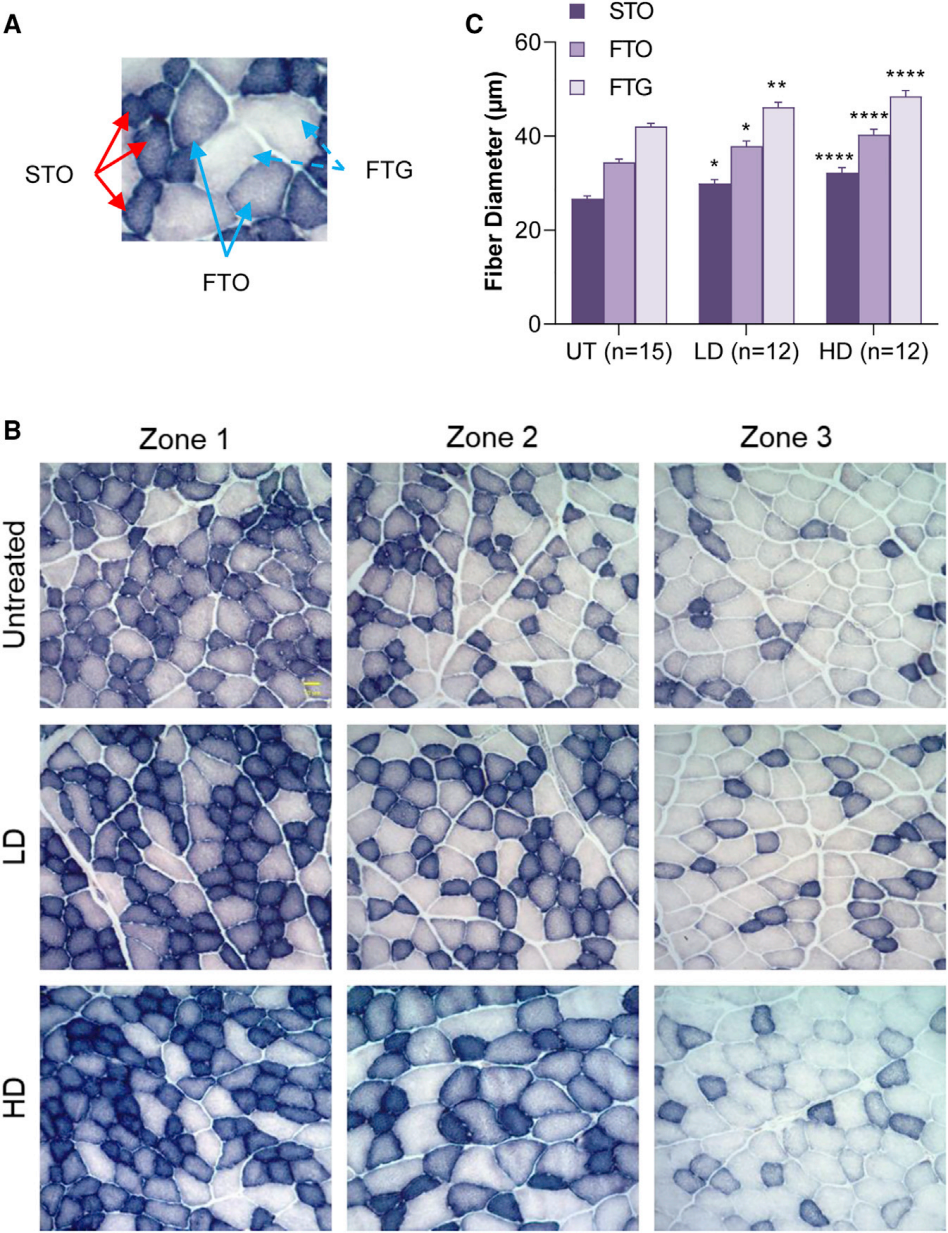
Muscle fiber size increase in CAPN3 KO mice with AAV.CAPN3 gene therapy (A) SDH staining showing muscle fiber types in TA muscle based on mitochondrial content: fatigue-resistant slow-twitch oxidative (STO; dark) or type1 fibers, fast-twitch oxidative (FTO; intermediate) fibers with intermediate staining intensity, and fast-twitch glycolytic (FTG; light) fibers with the lightest staining intensity. (B) Representative images from TA muscle showing three different zones (deep, intermediate, and superficial; designated zones 1, 2, and 3) from the UT, LD (3 × 10^12^ vg), and HD (6 × 10^12^ vg) treatment cohorts. (C) Fiber size measurements, done on images derived from 3 zones of TA muscle, showed significant increases for all fiber types (STO, FTO, and FTG) in the LD and HD cohorts compared with the UT CAPN3 KO group. Each bar represents the mean ± SEM of 12–15 mice in each group. ∗p < 0.05, ∗∗p < 0.01, ∗∗∗∗p < 0.0001 (compared with UT), two-way ANOVA, Tukey’s multiple comparisons test. Scale bar, 30 μm in the UT zone (applies to all).

We quantified the effects of CAPN3 gene therapy in CAPN3 KO mice upon muscle fiber size and fiber type composition 20 weeks after gene injection in distal and proximal lower limb muscles (gastrocnemius, *tibialis anterior*, and quadriceps) as well as in triceps muscle from upper limbs using succinic dehydrogenase (SDH) staining, which delineates fibers with high mitochondrial content; i.e., fatigue-resistant slow-twitch oxidative (STO) or type 1 fibers, fast-twitch oxidative (FTO) fibers with intermediate staining intensity, and fast-twitch glycolytic (FTG) fibers with the lightest staining intensity (Figure 4A). Fiber size measurements were done on representative images from 3 zones of the muscle (deep, intermediate, and superficial, designated zones 1, 2, and 3, respectively), as illustrated in the *tibialis anterior* muscle from the old age group (Figures 4B and 4C; Table S3). Compared with the WT, overall muscle bulk and in particular the STO muscle fiber size of all fiber types were smaller in UT *Capn3*-null muscles, corroborating previous reports,^11^^,^^21^ with the exception of the triceps, the only upper limb muscle we analyzed in the older age group in which UT STO fiber size was not different from the WT (Tables S3–S14). CAPN3 gene therapy in KO mice resulted in a significant fiber size increase in all fiber types from *tibialis anterior* muscle in the LD and HD cohorts compared with the UT cohort (Table S3). Increases in mean fiber size were observed in other muscles from old and young age groups of CAPN3 KO mice (Tables S3–S8; Figures S1–S5).

Further analysis of data for females and males suggested that the improvements in the treadmill test correlated with muscle remodeling, a switch to fatigue-resistant oxidative fibers in females along with an increase in fiber size, and a significant increase in fiber diameter that was most prominent in STO fibers in males. Changes in fiber size and fiber type distribution of STO, FTO, and FTG (as percent of total) observed in the treated cohorts in comparison with UT counterparts are represented in Figure S6, which shows the presence of a trend or significant decrease in FTG fibers in all *tibialis anterior* and gastrocnemius samples from female CAPN3 KO mice in both age groups. The decrease in the FTG subgroup was reflected as a switch to the oxidative fiber phenotype, resulting in an increase in STO or FTO fibers. In the old group, LD and HD treatment lead to an increase in STO fibers in both muscles, whereas in the young group, there were few exceptions. The STO percentage did not increase in LD-treated *tibialis anterior* and gastrocnemius muscles at both doses, but the FTO subtype increased in all (Tables S9–S12; Figures S7–S10). An overall trend of switching to STO with CAPN3 replacement was also present in LD- and HD-treated quadriceps muscle from females in the old group (Figures S11; Table S13). Triceps muscle responded to treatment with an increase in FTO fibers in females at LD and HD, whereas an STO increase was observed only in the LD cohort (Figure S12; Table S14). Fiber type diameter (mean ± SEM μm, derived from the mean of measurements made individually in each mouse) and fiber type distributions (derived from percent distribution in each mouse) with age-matched WT data are detailed in Tables S3–S14 and Figures S1–S12. Collectively, histological analyses of skeletal muscle in CAPN3 KO mice 20 weeks after gene delivery reflected effects of CAPN3 replacement by remodeling of muscle and an overall switch to oxidative fibers in young and old age groups along with fiber size increase.

#### Vector copy number analysis

As part of the efficacy analysis of *CAPN3* gene transfer, we assessed the biodistribution of the vector among tissues by analyzing the vector genome copies in muscles (*tibialis anterior*, gastrocnemius, quadriceps, and triceps) and the internal organs collected from LD and HD cohorts of the old age group of CAPN3 KO mice 20 weeks after treatment.

Biodistribution of AAVrh74.tMCK.CAPN3 in CAPN3 KO mice had broad tropism and efficiently targeted skeletal muscles. We found that the highest vector genome copy number was present in the liver, as expected, following systemic vector delivery (Figure S13A). Vector genome distribution was variable in organs and muscles in a dose-dependent manner. HD delivery led to a 59.6% increase in skeletal muscle vector genome levels (p < 0.0001) compared with LD delivery (Figure S13B).

### *CAPN3* expression and functional correlation

**Figure 5:**
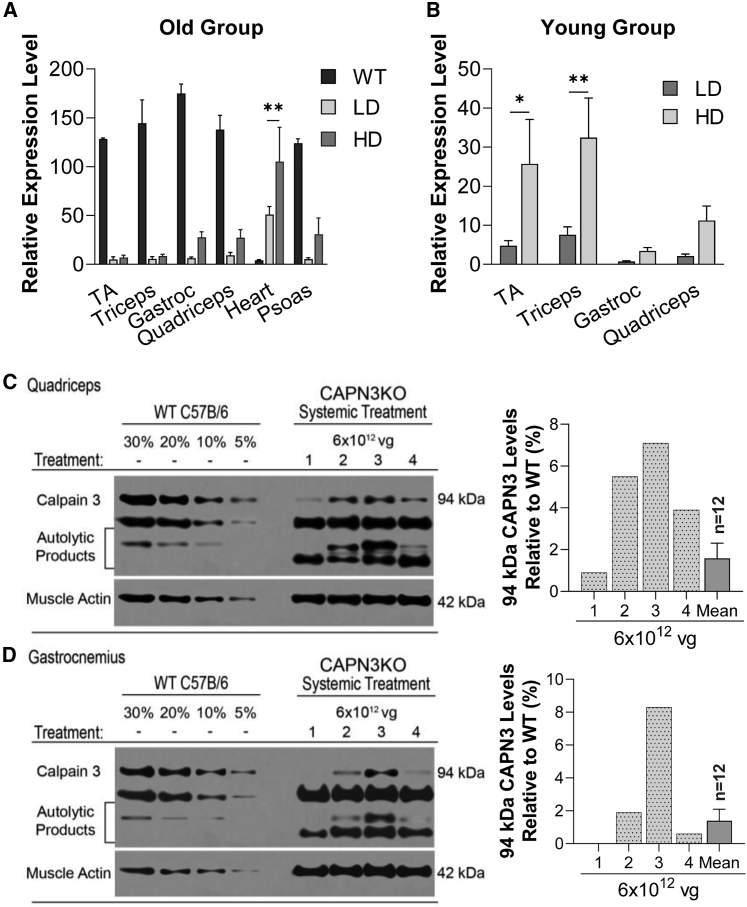
Muscle tissue expression of *hCAPN3* at mRNA and protein levels in CAPN3 KO mice 20 weeks after gene delivery (A and B) Relative expression levels of *hCAPN3* in several skeletal and cardiac muscle tissue samples from the old (A) and young age groups of CAPN3 KO mice (B). A dose-dependent increase in *CAPN3* mRNA transcripts was observed in the HD (6 × 10^12^ vg, n = 12) treatment cohort compared with the LD (3 × 10^12^ vg, n = 12) cohort for both age groups. GAPDH was used as the reference gene. Data are represented as mean ± SEM. ∗p < 0.05, ∗∗p < 0.01, two-way ANOVA, Sidak’s multiple comparison test. (C and D) Representative western blot images from 4 mice show variable amounts of 94-kDa CAPN3 protein in quadriceps (C) and gastrocnemius (D) muscles from the HD cohort of the old age group. A calibration curve ranging from 15–2.5 μg (30%–5%) total protein from the corresponding UT WT C57BL/6 reference muscles was used for semiquantitative analysis of AAV-mediated CAPN3 levels in the corresponding treated CAPN3 KO mouse muscles. CAPN3 levels were detected using the CALP-12A2 antibody, which recognizes epitopes in muscle extracts from human and mouse. Assay sensitivity was optimized to reliably detect the CAPN3 protein in amounts as low as 2.5% of the WT control level. CAPN3 protein levels in the quadriceps (C) and gastrocnemius (D) are quantified as percent of the WT and represented individually as bar graphs, and the mean levels of all HD samples (n = 12) are given as mean ± SEM.

Tissue expression of *hCAPN3* was carried out at mRNA and protein levels in skeletal and cardiac muscle samples collected from UT and LD- or HD-treated CAPN3 KO cohorts 20 weeks after AAVrh74.tMCK.CAPN3 vector delivery as well as in muscles from age-matched WT mice. Using qPCR, sustained *CAPN3* mRNA expression was observed in four representative muscles from hindlimbs and forelimbs (*tibialis anterior*, gastrocnemius, quadriceps, and triceps) and the heart in the CAPN3 KO model. *CAPN3* mRNA levels were variable among muscles/animals in a dose-dependent manner. Skeletal muscles of young and old age groups that received an HD of the vector showed higher *CAPN3* mRNA levels (difference in young age group: 14.4%, p = 0.001; in the old age group: 11.0%, p < 0.001) compared with LD cohorts in both age groups (Figures 5A and 5B). There was no significant difference in skeletal muscle *CAPN3* mRNA expression related to the animal’s treatment age (mean difference < 2%, p = 0.72, unpaired t test).

Western blot analyses showed measurable amounts of full-length, 94-kDa CAPN3 protein in quadriceps and gastrocnemius muscles of HD-treated mice, as shown in the older age group (Figures 5C and 5D). These two muscles had the highest CAPN3 expression levels. Protein levels were also variable among muscles/animals and did not directly correlate with mRNA levels. In the LD cohort, full-length, 94-kDa protein was below the limit of detection in all except a gastrocnemius sample, with the highest mRNA expression suggesting a mRNA level threshold for detecting the 94-kDa CAPN3 protein band in muscles with a *Capn3*-null background.

**Figure 6:**
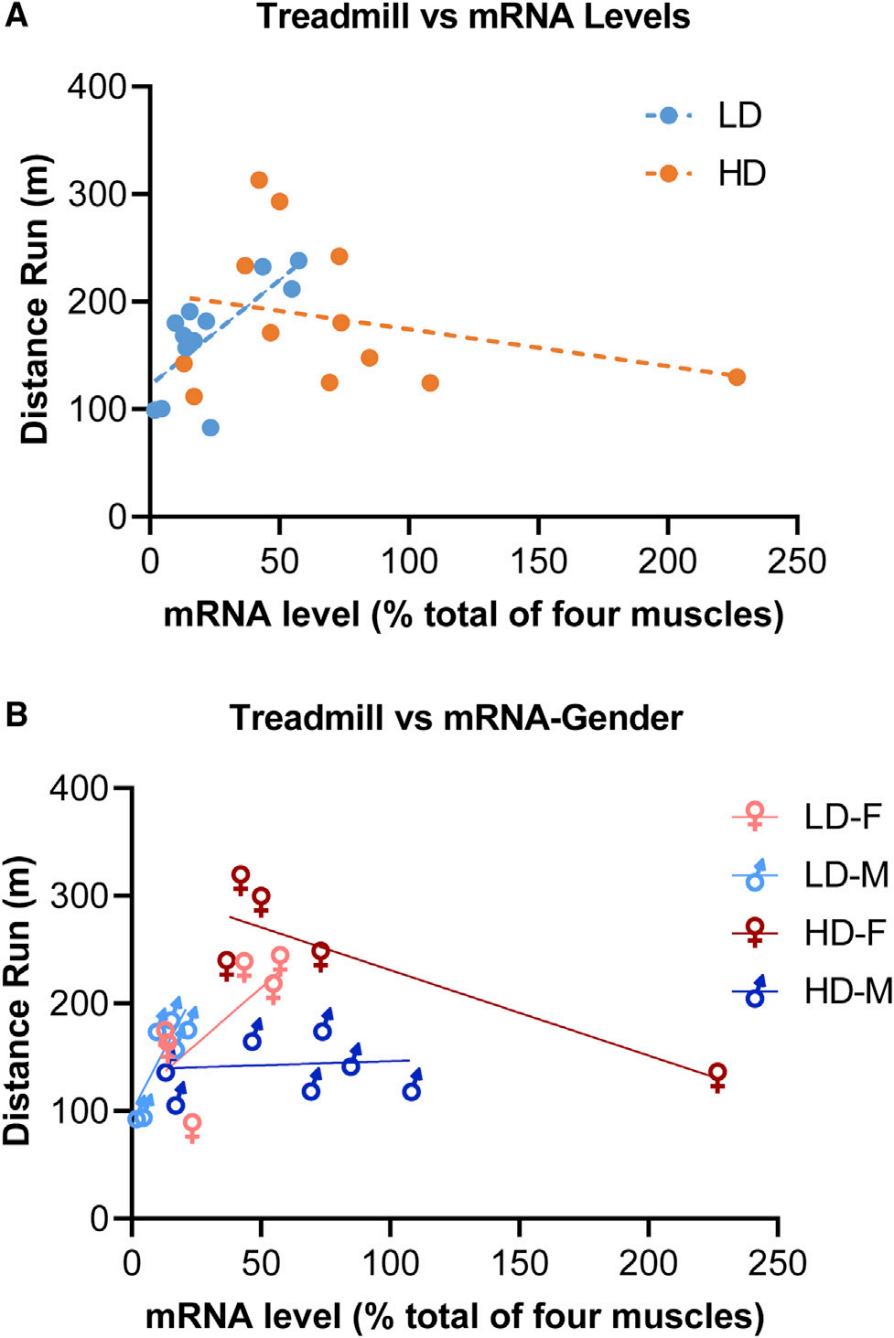
Correlation of *CAPN3* expression levels with treadmill performance (A) Scattergram showing a correlation between the total mRNA levels from four representative muscles (TA, gastrocnemius, quadriceps, and triceps) from the old age group correlating with the run-to-exhaustion test in the LD (3 × 10^12^ vg, n = 12) cohort (R^2^ = 0.5192, p = 0.0082, linear regression analysis). In the HD cohort (6 × 10^12^ vg, n = 12), no correlation was seen between mRNA levels and run-to-exhaustion test performance. (B) The mRNA level window for the best treadmill performance favored females from the HD and LD cohorts.

We next sought to determine whether *CAPN3* mRNA levels correlated with functional improvements in treadmill performance. For this analysis, a total mRNA percent value for each animal was calculated, derived from the summation of relative *CAPN3* mRNA levels of the gastrocnemius, *tibialis anterior*, quadriceps, and triceps muscles. We reasoned that, because all four muscles are exerted during the treadmill run, the total mRNA percent value may serve as a functional performance indicator. For the old age group, the total mRNA levels from all four muscles suggested a correlation with the run-to-exhaustion test in the LD cohort (R^2^ = 0.5192, p = 0.0082), whereas in the HD cohort, no correlation was seen between mRNA levels and run-to-exhaustion test performance (Figure 6A). Mice with high performance in the LD cohort overlapped with the best performers of the HD cohort, suggesting that mRNA levels exceeding a threshold might not further improve performance. These results favor an mRNA threshold effect required to improve performance. The occurrence of a window of mRNA levels for the best treadmill performance indicated an effect of sex difference, favoring females (Figure 6B). The correlation between treadmill performance and mRNA levels observed in the old group was not evident in the young, suggesting that gender- and age-related molecular changes might affect the severity of disease manifestation and treatment efficacy.

### PGC1α expression levels and muscle remodeling

So far, we have shown that CAPN3 gene therapy resulted in a fiber type switch from FTG to STO fatigue-resistant fibers, associated with improvements in run-to-exhaustion endurance performance, favoring females. These observations prompted further studies to explore whether there were sex-dependent alterations in expression levels of peroxisome proliferator-activated receptor γ coactivator 1 alpha (PGC1α), an important transcriptional coactivator of mitochondrial biogenesis and respiration.^31^ Previous studies have shown that PGC1α drives formation of fatigue-resistant STO muscle fibers.^32^ Moreover, PGC1α regulates the expression and activity of the orphan nuclear receptor estrogen-related receptor alpha (ERRα).^33^ We have shown previously that *Pgc1a* levels decreased in CAPN3 KO mice during the regeneration process compared with WT muscle.^21^ Here we analyzed the expression levels of *Pgc1a* in response to CAPN3 gene therapy in muscles from the HD and LD treatment cohorts of old and young groups of CAPN3 KO and age-matched WT mice.

**Figure 7:**
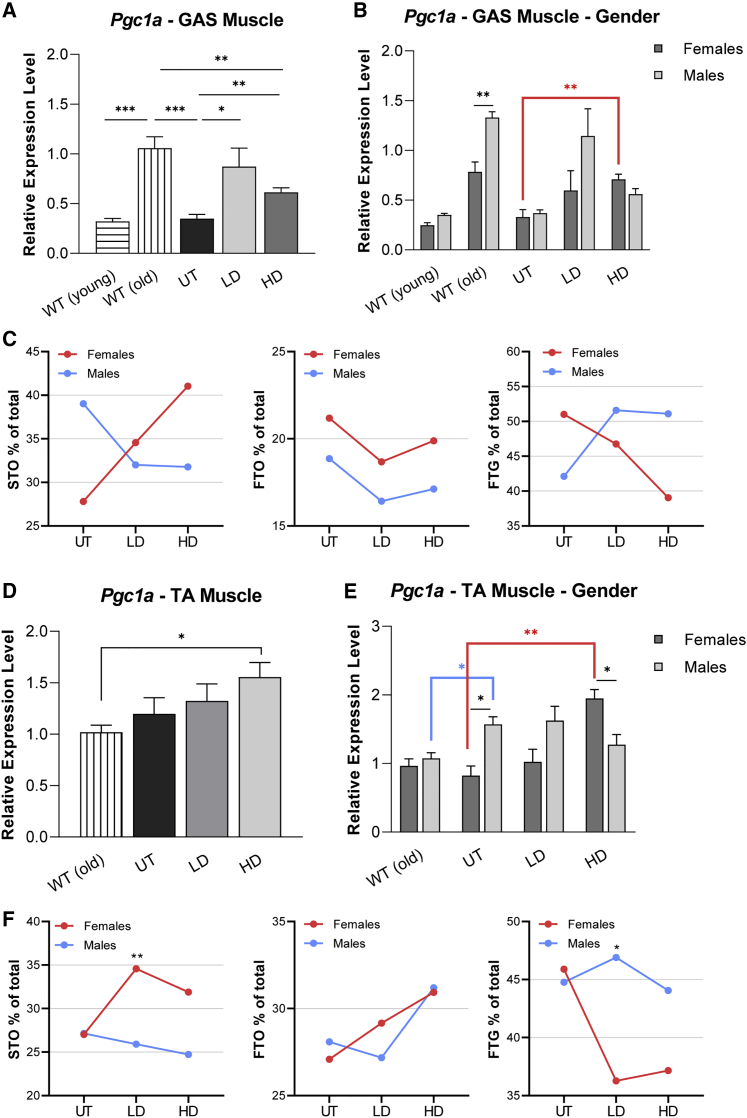
Relative *Pgc1a* expression levels in muscle and its correlation with fiber type remodeling (A, B, D, and E) Relative expression levels of *Pgc1a* in gastrocnemius (GAS; A and B) and TA (D and E) muscles from the old age group of CAPN3 KO (UT, n = 8; LD, n = 12; HD, n = 12) and age-matched WT (n = 8) mice 20 weeks after *hCAPN3* gene delivery are shown as both sexes combined (A and D) and separated (B and E). (C and F) Line graphs showing the percent changes in fiber type (STO, FTO, and FTG) distribution in the same muscles, GAS (C) and TA (F), from the LD and HD treatment cohorts. Data are represented as mean ± SEM. ∗p < 0.05, ∗∗p < 0.01, ∗∗∗p < 0.001, unpaired t test. A switch to STO (as percent increase) in the HD and LD cohorts was observed only in females (C and F) even though LD males showed higher *Pgc1a* expression than females (B and E).

When gastrocnemius muscles from two different ages of WT controls were compared, we identified an increase in relative expression levels of *Pgc1a* at 10 months of age, with males showing significantly higher *Pgc1a* expression than females compared with samples from 4- to 6-month-old WT mice showing no gender difference (Figures 7A and 7B). In contrast, *Pgc1a* expression was significantly lower in UT CAPN3 KO mice of both sexes at 10 months of age, suggesting that *Capn3*-null muscle failed to show this increase, likely an age-related compensatory change that occurred in WT mice at this age (Figures 7A and 7B). Gene therapy in the LD and HD cohorts significantly increased *Pgc1a* transcripts compared with the UT cohort (Figure 7A). Increases in *Pgc1a* transcripts were seen in both genders toward normalization (Figure 7B); however, the fiber type switch to STO occurred only in females of both treatment cohorts (Figure S1; Figure 7C).

We then investigated whether changes in *Pgc1a* expression vary between posterior and anterior compartment muscles in response to AAV.CAPN3 gene therapy. In the *anterior tibialis*, both sexes combined, we found no statistically significant changes in *Pgc1a* expression between WT, LD, and UT cohorts, but in the HD cohort there was an increase in *Pgc1a* transcripts compared with the WT (Figure 7D). Contrary to the gastrocnemius muscle of the WT, relative Pgc*1a* expression in the *tibialis anterior* from 10-month-old WT mice was about 50% lower and did not show sex differences (Figures 7D and 7E). UT CAPN3 KO males displayed significantly higher *Pgc1a* transcripts than WT males (Figure 7E). Interestingly, although there was no sex difference for *Pgc1a* expression in UT gastrocnemius, significantly increased levels were found in UT *anterior tibialis* muscle from males compared with females. Males in the LD treatment cohort showed a similar pattern as the UT cohort, having higher *Pgc1a* expression than females. We observed a reversal of this pattern in the HD cohort in favor of females having a 2.36-fold increase in *Pgc1a* transcripts compared with UT CAPN3KO females. Like in the gastrocnemius (extensor muscle), in the *tibialis anterior* (flexor muscle), a switch to STO (as percent increase) in the HD and LD cohorts was observed only in females (Figure 7F) even though LD males showed higher Pgc1a expression than females (Figure 7E). *Pgc1a* transcripts in these muscles from young CAPN3 KO mice did not differ from each other, but gastrocnemius muscle of LD males showed significantly higher *Pgc1a* expression than that of females in the same group and of males in the UT cohort (data not shown). These results suggest that females are responding to a *Pgc1a* increase in muscle more efficiently by converting the FTG to the STO fiber type. Another possibility is that the male response to *Pgc1a* increasing in muscle is not the same as in female muscle.

### Safety studies

**Figure 8:**
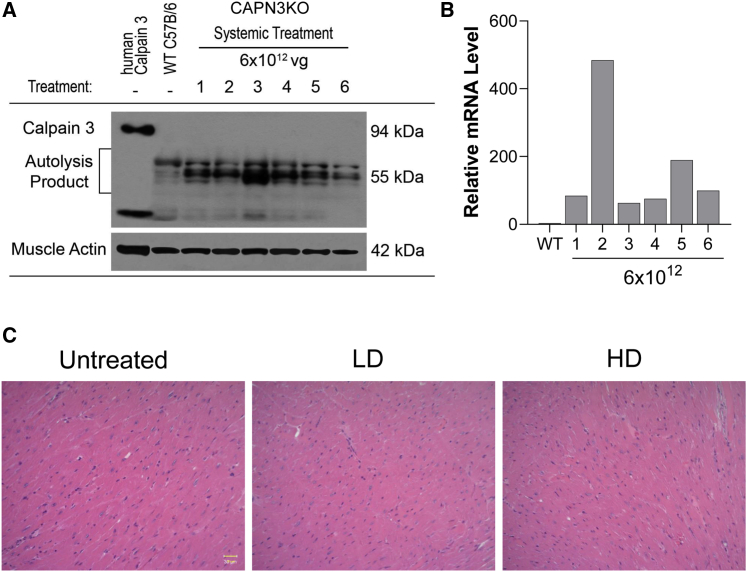
Histological and western blot analyses show no evidence of cardiotoxicity following systemic injection of the AAVrh7.4.tMCK.hCAPN3 vector (A) Western blot analysis of cardiac tissue from the HD (6 × 10^12^ vg) cohort 20 weeks after systemic injection of the AAVrh7.4.tMCK.hCAPN3 vector showed no detectable CAPN3 protein. A full-length, 94-kDa hCAPN3 band is present in human skeletal muscle as a positive control. (B) mRNA levels of mice corresponding to samples shown in the western blot, represented individually as bar graphs. (C) H&E-stained paraffin sections of the left ventricles from representative heart samples of CAPN3 KO mice 20 weeks after systemic injection of the LD (3 × 10^12^ vg) and HD revealed no histopathology, indicating no long-term toxicity. Muscle fiber necrosis, regeneration, or inflammation was not seen. Scale bar, 30 μm in the UT image (applies to all).

We conducted studies assessing the safety of the test article, including vector biodistribution, capsid immunogenicity, hematology, serum chemistry, and pathological review of multiple organ tissues. Biodistribution analysis showed the vector to be located globally; however, protein detection was limited to the target skeletal muscles; specifically, full-length, 94-kDa CAPN3 protein was below the limit of detection in western blot analyses of liver, kidney, or heart tissue (from all HD groups and the LD old group) despite the high vector genome load and mRNA levels observed in these tissues (Figure S13A). The absence of full-length, 94-kDa CAPN3 protein in heart samples from the HD cohort and corresponding variable amounts of mRNA are shown in Figures 8A and 8B. With the CALP-2C4 antibody, we detected the presence of autolysis products, suggesting that the full-length protein undergoes rapid degradation. Importantly, no adverse clinical signs of toxicity were observed throughout the 20-week study at either treatment dose. No gross abnormalities were noted, and no differences in animal or organ weights were observed in treatment groups as compared with UT CAPN3 KO controls. Microscopy evaluation of heart, lung, liver, kidney, spleen, gonads, diaphragm, stomach, pancreas, brain, inguinal lymph nodes, and skeletal muscle removed for histopathology observations by a third party, Vet Path Services, reported no treatment-related microscopic differences among the UT and treated groups at the 20-week study endpoint (data not shown, except heart in Figure 8C). Furthermore, hematology and serum chemistries showed no abnormal changes (data not shown), suggesting that ssAAVrh74.tMCK.CAPN3 gene therapy is an overall safe and effective treatment for LGMD2A.

## Discussion

We report here that systemic gene therapy using AAVrh74.tMCK.hCAPN3 rescued the phenotype in the LGMD2A/R1 mouse model and produced robust functional improvements supported by significant changes in isometric muscle contraction (maximum twitch response) and fatigue (maximum tetanic response) protocols as well as in histopathological parameters.

Moving forward to a clinical trial for LGMD2A/R1 from preliminary biopotency experiments requires consideration of several key questions: (1) the efficacy of the LD and HD to enhance muscle function and endurance in the CAPN3 KO model independent of age, (2) the influence of gender differences on treatment response, (3) prevention of cardiotoxicity following long-term muscle-specific expression of CAPN3, and (4) the correlation between full-length human CAPN3 protein or mRNA expression levels and muscle function and endurance in the *CAPN3* null-background. We tested these questions utilizing young and old age groups of both genders in dose-ascending experiments in CAPN3 KO mice following systemic delivery of the AAVrh74 vector containing *hCAPN3* cDNA.

Previously, a study using rAAV2/1-mediated CAPN3 transfer in a *Capn3*-deficient murine model reported that the model demonstrated efficient transgene expression in skeletal muscle. RNA and protein levels were restored, demonstrating proteolytic activity and improvement of histological features 1 month after IM gene transfer, resulting in correct sarcomere localization of the transgene product.^27^ More importantly, based on western blot results, it was concluded that even a low amount of CAPN3 expressed in skeletal muscle was sufficient to attenuate the histopathological phenotype of *Capn3*-null mice.^28^ Supporting these observations, our study showed a muscle fiber size increase and fiber type remodeling accompanied by improvement in function at equal HD and LD levels. In these experiments, however, full-length, 94-kDa CAPN3 bands were below the limit of detection in four representative muscles (except in one gastrocnemius sample with the highest mRNA level detection) following LD systemic delivery of AAV.CAPN3; in these muscles, the HD cohort showed variable amounts of detectable protein (in the quadriceps, about 1%–7% of the WT), and we repeatedly observed higher levels of autolytic products in HD-treated mice. We speculate that the nascent transgene product undergoing a sequential activation process^5^ is utilized rapidly because of higher demand for CAPN3 in the null background producing more autolytic fragment, and in WT mice there is in an equilibrium of CAPN3 protein, resulting in more stable full-length protein. Because LD mice received less vector and therefore produced smaller amounts of CAPN3 protein, all CAPN3 products are utilized without reaching a level of accumulation. In contrast, we found sustained *CAPN3* mRNA expression in these hindlimb and forelimb muscles in treatment cohorts 20 weeks after gene delivery. Concurrently, the *CAPN3* mRNA levels were variable in a dose-dependent manner. These observations indicate that the amount of detected CAPN3 protein cannot be used to assess the efficiency of gene transfer in future human clinical trials. Moreover, we cannot rely on protein detection because some *CAPN3* mutations have been shown to result in normal/reduced expression of the full-length protein.^34^^,^^35^ Accordingly, no direct correlation has been observed between the amount of calpain and the severity of the phenotype.^36^

As expected, following systemic delivery, we found variable transgene expression among muscles/animals in a dose-dependent manner, favoring large muscle groups, in CAPN3 KO mice. Moreover, a correlation between combined *CAPN3* mRNA levels from four muscles and run-to-exhaustion test results suggested the presence of a window of mRNA levels for the best treadmill performance, favoring the females. Within this window, mice with the best performance in the LD cohort overlapped with the best performers of the HD cohort, suggesting that mRNA levels exceeding a threshold might not further improve function. Although there was a correlation between total mRNA levels and treadmill performance in the LD cohort, no correlation was found in the HD cohort. Gender difference did not influence treadmill performance in young CAPN3 KO mice, and the correlation between treadmill performance and mRNA levels observed in the old group was not evident, suggesting that gender- and age-related molecular changes might affect the severity of disease manifestation and treatment efficacy. Supporting this, heel cord contractures as an age-related phenotype, developed only in males from the old age group, were prevented with HD treatment. In contrast, all UT males and 4 of 6 males in the LD cohort displayed varying degrees of heel cord contractures at the endpoint.

Studies of human skeletal muscle have revealed the presence of a sex difference in fiber type composition, with males having a larger type 2 area and, thus, a more glycolytic phenotype, whereas females show a higher percentage area of type I fibers with a more oxidative phenotype.^37^ These differences were also reflected in transcriptome analyses where males were enriched for many glycolytic mRNAs; in contrast, females were enriched for oxidative markers with robust enrichment in mitochondrial function, including the cofactor PGC1α.^38^, ^39^, ^40^ PGC1α is a major factor promoting glycolytic muscle fiber transformation to the oxidative muscle fiber type by upregulating the expression of oxidative muscle fiber-specific genes.^32^ In humans, expression of PGC1α has been shown to have a direct correlation with muscle fiber-type composition.^41^ Similar to these observations in human skeletal muscle, we found a greater proportion of fatigue-resistant STO fibers in females, reflecting normalization, compared with the WT following gene therapy in CAPN3 KO mice. This fiber type remodeling of muscle occurred with a switch to STO fibers from FTG, associated with increased *Pgc1a* expression. In contrast, males responded to *CAPN3* replacement with an overall fiber size increase only, which was most prominent for FTG fibers.

Our observations in 10-month-old WT mice suggest that there may be a muscle-specific gender difference in *Pgc1a* expression levels, with males showing significantly higher transcripts than females in the gastrocnemius compared with the *tibialis anterior*, where *Pgc1a* expression was found in half of the gastrocnemius muscle with no sex difference. Further studies are needed to explore whether functional, physiological, or histological changes occur that correlate with this change. Moreover, *Pgc1a* expression may show compensatory changes with aging as this study illustrated; at 10 months of age, *Pgc1a* expression in WT gastrocnemius was significantly higher than in young WT gastrocnemius. Considering reports of decreased *Pgc1a* expression in sarcopenic muscle from older species (2 years of age for rodents),^42^, ^43^, ^44^, ^45^ our findings of increased *Pgc1a* transcripts in 10-month-old WT muscle may represent a compensatory change during muscle aging before the sarcopenic state can be seen. Our results in this animal model of LGMD2A/R1 showed that AAV.CAPN3 gene therapy restores the relationship between *Pgc1a* expression and muscle fiber type and, therefore, functional endurance. In females, this bestows prominent fiber type remodeling, whereas in males, we see an increase in fiber size. It is important to re-emphasize that AAV.CAPN3 gene therapy effectively improved muscle performance, displaying unequivocal disease-modifying effects in males and females in both cohorts independent of dose.

The results of our safety studies provided important data for use of our vector to ensure muscle-specific expression of CAPN3 in null muscles and multiple organ tissues of this model, particularly cardiac tissue. In short- and long-term studies following vector delivery, we found no gross or histopathological abnormalities in the heart and no detectable protein bands in western blots. Our finding of lack of cardiac toxicity does not contradict the results of published reports demonstrating cardiac fibrosis in animals treated with the AAV9 vector delivering the desmin-hCAPN3 cassette.^28^ Cardiac fibrosis following desmin-C3 treatment has been observed in WT C57BL/6 and CAPN3 KO mouse models; however, no full-length, 94-kDa CAPN3 protein accumulated despite the evident pathology. These deleterious observations were not evident in mice treated with an AAV9-mediated expression cassettes driven by the CAPN3 endogenous promoter or by the miR206 promoter sequence with skeletal muscle-specific transcriptional activity.^28^ In our findings, human CAPN3 expression under control of the tMCK promoter (analogous to the skeletal muscle-specific miR206 promoter^46^) did not have observable cardiac impairment in CAPN3 KO mice. Histological sections were normal and lacked morbidity or sudden mortality as seen in the desmin-C3 mouse studies. An important finding in our study is that the safety profile remains, even in HD-treated animals (2.35 × 10^14^ vg/kg). Both AAV serotypes, the Rh74 used in our studies and the AAV9 used by Roudaut et al.,^28^ demonstrated cardiac tropism; however, even with delivery of higher systemic dosages of AAVrh74, no cardiac issues were encountered. Furthermore, the levels of cardiac *CAPN3* mRNA expression we observed did not cause toxicity in CAPN3 KO mice in the heart or any other tissues we studied.

In a recent study, although great work was done to demonstrate the role of the titin protein in the observed mouse cardiac toxicity with the AAV9-desmin-hCAPN3 construct but not in non-human primates,^47^ it was still underappreciated that the observed cardiomyopathy only occurs in the context of the desmin-C3 vector. Promotor selection and therapeutic utility rely on target tissue expression; however, it is equally critical to minimize potential adverse effects of off-target protein expression by limiting transgene expression to areas compatible with normal endogenous expression. Although robust skeletal muscle expression of CAPN3 was demonstrated, cardiac toxicity was an important consideration while developing our CAPN3 therapeutic vector and safety evaluations. In our study, despite observing cardiac transcriptional activity in treated animals, these elevated *CAPN3* mRNA levels did not lead to 94-kDa CAPN3 accumulation and were not evident even in the overexposed western blot films. Previous studies suggest a finely tuned and species-dependent regulatory mechanism directing CAPN3 patterns across distinct organs and tissues, including between the skeletal muscle and heart, during embryonic development as well as under normal physiological conditions.^47^, ^48^, ^49^, ^50^ It has been reported that CAPN3 RNA disappears from the ventricular compartment later in the embryonic heart, and although CAPN3 transcripts are present in the heart, the corresponding protein is not detected elsewhere than in skeletal muscle during the same stage of development.^50^ It is plausible that cardiac CAPN3 protein stability, turnover, and enzymatic activity are governed by diverse pathways, perhaps by direct action or convergent processes unique to the CAPN3 ortholog, tissue type, and physiological environment. The status of Ca^2+^ and Na^+^ concentrations, protein interactions with titin, or platform element for inhibition of autolytic degradation may appropriately modulate CAPN3 activity in the heart.^8^^,^^47^^,^^49^

Previous studies in CAPN3-null mice showed that impairment of muscle force and function becomes distinguishable at around 7–12 months without continuous worsening, and histological alterations become significant at 6 months of age.^51^ Although we treated mice at 1.5–2.5 and 5–6 months of age without symptoms, we found that mice at the endpoint (10–11 months), when predicted to be symptomatic with reduced muscle force and function, these parameters remained completely normal in our treated mice. This would not have been possible without early treatment. We also know from clinical studies of spinal muscular atrophy^52^ and Duchenne muscular dystrophy^53^ that we must treat before there are clinical features of disease to achieve normality. After many gene therapy trials, we have clearly learned that gene replacement therapy does not reverse disease, although it may prevent further deterioration or slow down progression; treatment only at the presymptomatic stage results in a virtual cure, maintaining the normal phenotype.^52^ Systemic administration of AAVrh74.tMCK.hCAPN3 in the LGMD2A/R1 mouse model significantly improved the functional, physiological, and histopathological parameters at the LD and HD independent of age at the time of vector administration and without cardiotoxicity. These translational studies are relevant for consideration of viral dose as well as baseline functional status in clinical trials; gender differences may also have implications for LGMD2A/R1.

## Materials and methods

### Animals, procedures, and treatment cohorts

Animals for this study were generated from our colony at The Abigail Wexner Research Institute at Nationwide Children’s Hospital from two breeding pairs of CAPN3 KO mice obtained from the University of California, Los Angeles (Dr. Melissa J. Spencer). Genotypes were established by PCR analysis of genomic DNA isolated from tail clips. All animal experiments were performed according to the guidelines approved by The Abigail Wexner Research Institute at Nationwide Children’s Hospital Animal Care and Use Committee (IACUC protocol ID:AR12-00014). The rAAVrh74.tMCK.CAPN3 vector was delivered systemically to CAPN3 KO mice via tail vein injection in two age groups: 6–10 weeks of age, representing the young group, and 20–24 weeks of age, representing the old group. Each age group was further divided into three cohorts according to the treatment dose. LD cohorts (n = 12) were injected with 3 × 10^12^ vg, and HD groups (n = 12) received 6 × 10^12^ vg of rAAVrh74.tMCK.CAPN3. Because of gender differences in weight at the time of injection, the calculated vg/kg corresponds to males receiving an LD of 1.02 × 10^14^ vg/kg and HD of 2.03 × 10^14^ vg/kg and females an LD of 1.33 × 10^14^ and HD of 2.66 × 10^14^ vg/kg. Doses were based on qPCR using a supercoiled plasmid standard. Age-matched UT control groups were injected with Ringer’s lactate (UT young, n = 13; UT old, n = 15). Each cohort included an approximately equal number of males and females. Treatment efficacy was tested 20 weeks after gene delivery using functional, physiological, and histopathological outcomes. Mice were euthanized by an overdose of xylazine/ketamine to harvest skeletal muscles (gastrocnemius, psoas, quadriceps, *tibialis anterior*, and triceps) for molecular and histopathological analyses. Blood and internal organs (brain, diaphragm, heart, kidneys, liver, lungs, lymph nodes, stomach, and spleen) were also collected from the old cohort for in-house toxicology studies. WT mice used in this study were strain matched to CAPN3 KO mice, which have the C57BL/6 background.

### Generation of the ss-pAAV.tMCK.CAPN3-Kan cassette and production of the rh74-AAV-hCAPN3 vector

An ssAAV expression cassette with the tMCK promoter and a kanamycin resistance sequence were designed in our laboratory. We cloned the human *CAPN3* open reading frame from a gene fragment (Eurofins Genomics, USA) downstream of the tMCK promoter, followed by a chimeric intron (human β-globin donor and immunoglobulin heavy chain acceptor)^54^ and a KOZAK sequence. The final cassette was sequenced before AAV production. AAV-hCAPN3 vectors with the rh74 serotype were produced and purified, and the physical titer of the encapsulated vector was determined by qPCR using a supercoiled plasmid standard^55^ by the viral vector core at Nationwide Children’s Hospital, Aliquoted vectors were kept at −80°C until use.

### Histological analysis of muscle

Gastrocnemius, *tibialis anterior*, quadriceps, and triceps muscles from HD and LD AAVrh74.tMCK.CAPN3 cohorts and Ringer’s lactate-injected CAPN3 KO mice (UT cohort) were removed, and 12-μm-thick cross-cryostat sections were subjected to SDH enzyme histochemistry to assess fiber type differentiation using the standard protocol established in our laboratory for muscle fiber type-specific diameter measurements, as described previously.^21^ Briefly, three images, each representing three different zones of muscle (deep, intermediate, and superficial) were photographed at 20× magnification using an Olympus BX41 microscope and SPOT camera (0.2434 mm^2^ area per section per zone, 0.7302 mm^2^ area per animal). This approach was chosen to capture the alterations in the oxidative state of fibers in each zone in response to treatment. Diameters of fatigue-resistant STO (dark), FTO (intermediate), and FTG (light) fibers were determined by measuring the shortest distance across the muscle fiber using Zeiss Axiovision LE4 software. The mean fiber diameter (mean ± SEM) was derived by combining all 3 fiber types, and fiber type percent contribution to the total was determined for each mouse from each treatment group. Data were derived from a total of 102 muscle samples from a total of 35 mice (3–4 muscles per mouse) in the old age group and a total of 36 muscle samples from a total of 18 mice (2 muscles per mouse) mice in the young age group (Figure 5). For the LD and HD treatment cohorts and the UT cohort of the old age group, a total of 8,946, 10,820 and 13,443 measurements were done, respectively. For the young cohort, a total of 1,736 and 2,809 fibers of the LD cohort, 2,111 and 2,731 fibers of the HD cohort, and 2,148 and 2,847 fibers of the UT cohort were measured for *tibialis anterior* and gastrocnemius, respectively.

### Run-to-exhaustion test

Mice were exercised to exhaustion on a treadmill (Columbus Instruments, Exer-6M treadmill) using a protocol adapted from Dr. Melissa J. Spencer’s lab at The University of California, Los Angeles.^19^ The treadmill was operated at a 4°–5° decline. Mice were acclimated to the treadmill for 3 days before data collection, with 10-min-long 7–10 m/min runs every day. Mice were run to exhaustion by increasing the treadmill speed 1 m/min each minute, starting at an initial velocity of 7 m/min. Lanes have a shock plate that pulses at a frequency of ∼3 Hz. Mice were considered “exhausted” when they were unable to re-engage the treadmill for 3 s after resting on the shock plate. Run duration was recorded and used to calculate the distance run.

### *In vivo* muscle contractility assay

This assay measures the aggregate torque produced by the plantar or dorsiflexor muscles of the lower limb and was done using a muscle physiology apparatus (Aurora Scientific, ON, Canada). The animal was anesthetized while its foot was attached to a pedal, called a ‘footplate,’ that was fastened to a dual-mode lever. When preparing the animal, the tibia was aligned perpendicular to the lever, and the hips were slightly raised. This permits torsion to occur in line with the axis of rotation. Using subcutaneous electromyography (EMG) electrodes, muscle contractility data were obtained by stimulating the tibial nerve to achieve gastrocnemius muscle torque around the ankle joint. Isometric contraction (maximum twitch response) and fatigue (maximum tetanic response) protocols were applied. The strengths of this assay include generation of physiologically relevant data, which enables longitudinal testing.

### DNA isolation and vector biodistribution analysis

Mice were sacrificed 20 weeks after administration of the AAVrh74 vector. Tissues were harvested, sectioned, and treated with proteinase K. The total genomic DNA was isolated manually with a protocol adapted from Damo et al.^56^ Absolute quantification of vector biodistribution was performed in duplicate using SYBR Green Master Mix and the 7500 Fast Real-Time PCR detection system (Applied Biosystems). A transgene-specific primer set was used at 0.25 μM final concentration using the sequences listed in Table S15. Results are reported as vector copies per microgram of genomic DNA.

### RNA isolation and mRNA expression

QIAzol lysis reagent was used to isolate RNA from select tissues. Total RNA was DNase treated and purified using the RNeasy Plus Universal Kit (QIAGEN). First-strand DNA was synthesized with the ProtoScript II cDNA Synthesis Kit (New England Biolabs). qRT-PCR for *CAPN3* and *PGC1α* was performed using the primer sets listed in Table S15. The 2^–ΔΔCt^ method was used for calculating relative expression. Expression data were normalized to mouse *GAPDH* mRNA levels and are shown as means of relative expression values obtained from duplicate samples and normalized to WT control levels (set at 1) in conjunction with the standard error of the mean and are presented in a graph format.

### Assessment of CAPN3 (full-length, 94-kDa) protein by western blot

From each tissue, fresh-frozen serial tissue sections were taken for protein preparation. Samples were harvested in buffer (125 mM Tris-HCl buffer [pH 6.8], 4% SDS, 5% glycerol, and 4 M urea) containing protease inhibitor (Roche Diagnostics). Insoluble material was discarded following high-speed centrifugation at 4°C. The protein concentration was measured using the Lowry assay (RC DC, Bio-Rad). Semiquantitative western blot analysis was performed 20 weeks after treatment by comparing vector-mediated CAPN3 with CAPN3 KO control and WT endogenous CAPN3 tissue levels. Muscle proteins (15, 10, 5, and 2.5 μg for the WT control curve and 50 μg for all other lanes) were separated by reducing SDS-PAGE on a 3%–8% Tris acetate gradient gel (NuPage, Invitrogen) and subsequently immobilized by electrotransfer onto polyvinylidene fluoride (PVDF) membranes (Amersham Biosciences). Monoclonal antibodies raised against CAPN3 (NCL-CALP-12A2 [1:40, Leica] or NCL-CALP-2C4 [1:20, Leica]) and actin (NCL-L-MSA [Leica]) were used for immunodetection. Bound antibodies were detected by horseradish peroxidase-conjugated secondary sheep anti-mouse immunoglobulin G (IgG; 1:9,000, GE Healthcare) and enhanced chemiluminescence (ECL) reagent (Amersham Biosciences). Densitometry was measured using ImageQuant TL.

### Statistical analysis

For comparisons between treatment groups, statistical analyses were performed in GraphPad Prism 9.0 software. Two-tailed Student’s t test, one-way ANOVA with Tukey’s multiple comparisons test, two-way ANOVA with Sidak’s multiple comparisons test, or linear regression analysis were performed when applicable, and the significance level was set at p ≤ 0.05. The tests that met the best assumptions of the data were chosen. Results are given as mean ± SEM for all experiments, and the number of animals is mentioned in the figure legends along with the statistical analysis performed.

## Supplemental information

Document S1. Figures S1–S13 and Tables S1–S15

Document S2. Article plus supplemental information
