# Supplementary material for: Systemic delivery of AAVrh74.tMCK.hCAPN3 rescues the phenotype in a mouse model for LGMD2A/R1
Source: Mol Ther Methods Clin Dev. 2021 Jun 24;22:401–14. doi: 10.1016/j.omtm.2021.06.010 (PMC8413669; doi:10.1016/j.omtm.2021.06.010)
Supplement: Document S1. Figures S1–S13 and Tables S1–S15 [file mmc1.pdf]

OMTM, Volume *22*

Supplemental information

Systemic delivery of AAVrh74.tMCK.hCAPN3 rescues the phenotype in a mouse model for LGMD2A/R1

Zarife Sahenk, Burcak Ozes, Darren Murrey, Morgan Myers, Kyle Moss, Mehmet E. Yalvac, Alicia Ridgley, Lei Chen, and Jerry R. Mendell

Table S1. Fiber size analysis of the tibialis anterior muscle from CAPN3KO mice at 4 weeks post-gene delivery and age-matched WT mice

	Untreated (n=3)		Low Dose (n=4)		High Dose (n=3)		WT (n=3)	
	**Number**	**Diameter (µm)**	**Number**	**Diameter (µm)**	**Number**	**Diameter (µm)**	**Number**	**Diameter (µm)**
**STO**	214.5 ± 32.0	27.15 ± 0.7	116.0 ± 11.9	30.65 ± 0.6	147.0 ± 20.1	31.70 ± 1.6	177.3 ± 46.1	30.02 ± 1.2
**FTO**	91.0 ± 6.7	35.4 ± 1.3	111.8 ± 7.4	40.78 ± 0.8*	115.0 ± 30.6	38.82 ± 0.9	92.7 ± 15.1	40.66 ± 2.7*
**FTG**	113.8 ± 7.6	39.67 ± 1.6	100.8 ± 7.1	44.11 ± 0.8	75.3 ± 4.1	45.83 ± 1.1*	91.7 ± 13.3	46.07 ± 2.3**
**All fiber**	328.5 ± 8.4	32.47 ± 1.5	328.5 ± 8.4	38.18 ± 0.2	337.3 ± 29.6	37.27 ± 1.1	361.7 ± 43.1	37.00 ± 2.2

Data represented as mean ± SEM. *p<0.05, **p<0.01 (compared to UT), Two-way ANOVA, Tukey's multiple comparisons test.

Table S2. Experimental cohorts of CAPN3KO mice included in the study.

**Groups**	**Age at treatment**	**Treatment cohorts**	**Treatment Dose**	**Average Treatment Dose (vg/kg)**	**Total # of mice**	**Female**	**Male**	**Treatment Duration**	**Age at End Point**
Young	6-10 wks	Low dose	$3x10^{12}$ vg	$1.17x10^{14}$	12	6	6	20 wks	26-30 wks
		High Dose	$6x10^{12}$ vg	$2.35x10^{14}$	12	6	6		
		Untreated	Ringer's Lactate	Ringer's Lactate	13	7	6		
Old	20-24 wks	Low dose	$3x10^{12}$ vg	$1.17x10^{14}$	12	6	6	20 wks	40-44 wks
		High Dose	$6x10^{12}$ vg	$2.35x10^{14}$	12	5	7		
		Untreated	Ringer's Lactate	Ringer's Lactate	15	8	7		

Table S3. Fiber size analysis of the tibialis anterior muscle from old CAPN3KO mice at 20 weeks post-gene delivery and age-matched WT mice.

	Untreated (n=15)		Low Dose (n=12)		High Dose (n=12)		WT (n=8)	
	**Number**	**Diameter (µm)**	**Number**	**Diameter (µm)**	**Number**	**Diameter (µm)**	**Number**	**Diameter (µm)**
**STO**	87.2 ± 8.3	26.71 ± 0.6	92.0 ± 6.6	29.96 ± 0.8**	85.3 ± 11.5	32.24 ± 1.0****	91.6 ± 10.1	30.49 ± 1.0**
**FTO**	86.1 ± 4.5	34.48 ± 0.6	86.0 ± 4.9	37.88 ± 1.0**	91.3 ± 6.2	40.31 ± 1.1***	66.3 ± 3.5	36.33 ± 1.1$^{\$}$
**FTG**	142.0 ± 6.1	42.08 ± 0.6	126.0 ± 7.7	46.21 ± 0.9**	119.9 ± 7.0$^{\#}$	48.52 ± 1.1****	162.5 ± 5.7	41.54 ± 2.0$^{\#\#\$\$\$}$
**All Fibers**	315.3 ± 13.8	35.85 ± 0.5	304.0± 8.9	38.96 ± 0.7**	296.5 ±17.9	41.39 ± 1.0****	320.4 ± 13.6	37.23 ± 0.9$^{\$}$

Data represented as mean ± SEM. *p<0.05, **p<0.01, ***p<0.001, ****p<0.0001 (compared to UT), $^{\#}$p<0.05, $^{\#\#}$p<0.01 (compared to LD), $^{\$}$p<0.05, $^{\$\$}$p<0.01 (compared to HD), unpaired t-test.

For the graph, please see Figure 4C.

Table S4. Fiber size analysis of the triceps muscle from old CAPN3KO mice at 20 weeks post-gene delivery and age-matched WT mice

	Untreated (n=15)		Low Dose (n=12)		High Dose (n=12)		WT (n=8)	
	**Number**	**Diameter (µm)**	**Number**	**Diameter (µm)**	**Number**	**Diameter (µm)**	**Number**	**Diameter (µm)**
**STO**	81.9 ± 13.0	26.52 ± 0.6	82.0 ± 8.8	28.30 ± 0.9	72.1 ± 7.7	29.37 ± 1.1*	72.4 ± 5.2	26.22 ± 0.7
**FTO**	68.9 ± 5.6	37.17 ± 1.0	76.9 ± 6.6	38.96 ± 1.0	71.3 ± 6.0	38.36 ± 1.4	58.8 ± 3.1	33.32 ± 0.7*$^{\#\#\$}$
**FTG**	139.7 ± 11.2	48.80 ± 1.3	137.8 ± 4.3	50.68 ± 1.2	146.4 ± 7.7	50.36 ± 1.3	188.9 ± 8.8*$^{\#\#\#\$\$}$	43.50 ± 1.1*$^{\#\#\$\$}$
**All Fibers**	290.5 ± 25.4	41.02 ± 1.3	296.4 ± 15.4	41.71 ± 1.4	289.8 ± 12.8	42.16 ± 1.3	320.0 ± 14.5	37.70 ± 0.8$^{\$}$

Data represented as mean ± SEM. *p<0.05, **p<0.01 (compared to UT), $^{\#}$p<0.05, $^{\#\#}$p<0.01 (compared to LD), $^{\$}$p<0.05, $^{\$\$}$p<0.01 (compared to HD), unpaired t-test.

Table S5. Fiber size analysis of the gastrocnemius muscle from old CAPN3KO mice at 20 weeks post-gene delivery and age-matched WT mice

	Untreated (n=8)		Low Dose (n=5)		High Dose (n=6)		WT (n=8)	
	**Number**	**Diameter (μm)**	**Number**	**Diameter (μm)**	**Number**	**Diameter (μm)**	**Number**	**Diameter (μm)**
**STO**	102.9 ± 8.3	26.41 ± 0.4	118.0 ± 8.0	26.91 ± 0.3	123.3 ± 6.9	29.50 ± 0.4**##	101.9 ± 6.8	29.96 ± 0.7**#
**FTO**	65.8 ± 5.6	35.02 ± 0.9	62.0 ± 4.3	33.82 ± 0.6	61.3 ± 4.1	36.90 ± 0.2#	48.5 ± 2.6	34.07 ± 0.7$
**FTG**	148.1 ± 5.2	43.25 ± 0.8	168.2 ± 5.7	42.09 ± 0.7	137.7 ± 3.4#	44.66 ± 0.7	170.5 ± 6.3*$$$	41.19 ± 1.0$
**All Fibers**	316.8 ± 12.1	36.18 ± 0.74	348.2 ± 8.7	35.37 ± 0.4	322.3 ± 6.9	37.35 ± 0.4#	320.9 ± 9.7	36.51 ± 0.8

Data represented as mean ± SEM. *p<0.05, **p<0.01 (compared to UT), #p<0.05, ##p<0.01 (compared to LD), $p<0.05, $$p<0.01 (compared to HD), unpaired t-test.

Table S6. Fiber size analysis of the quadriceps muscle from old CAPN3KO at 20 weeks post-gene delivery and age-matched WT mice

	Untreated (n=7)		High Dose (n=7)		WT (n=7)	
	**Number**	**Diameter (μm)**	**Number**	**Diameter (μm)**	**Number**	**Diameter (μm)**
**STO**	52.0 ± 5.2	26.34 ± 1.2	56.0 ± 13.4	30.27 ± 1.5	57.6 ± 9.6	31.66 ± 1.0**
**FTO**	44.4 ± 4.7	34.63 ± 1.7	50.9 ± 5.4	35.67 ± 1.5	39.0 ± 3.7	37.59 ± 0.8
**FTG**	169.7 ± 11.0	44.99 ± 1.5	157.4 ± 9.9	46.77 ± 1.8	162.0 ± 9.0	46.56 ± 1.4
**All Fibers**	232.9 ± 34.4	39.59 ± 1.5	264.3 ± 24.2	41.50 ± 1.9	258.6 ± 16.4	42.02 ± 1.3

Data represented as mean ± SEM. **p<0.01 (compared to UT), unpaired t-test.

Table S7. Fiber size analysis of the tibialis anterior muscle of CAPN3KO mice from young age group at 20 weeks post-gene delivery

	Untreated (n=6)		Low Dose (n=6)		High Dose (n=6)	
	**Number**	**Diameter (µm)**	**Number**	**Diameter (µm)**	**Number**	**Diameter (µm)**
**STO**	67.0 ± 8.3	24.29 ± 0.5	48.2 ± 5.0	27.47 ± 0.4**	94.0 ± 5.9$^{\#\#}$	27.13 ± 0.9
**FTO**	75.2 ± 4.6	29.06 ± 0.6	74.8 ± 6.0	33.69 ± 0.4**	88.7 ± 6.9	33.35 ± 1.0*
**FTG**	215.8 ± 6.2	38.40 ± 0.7	157.5 ± 6.0**	44.27 ± 0.5**	169.2 ± 7.9*	41.97 ± 1.3
**All Fibers**	358.0 ± 14.0	33.95 ± 0.8	289.3 ± 8.8*	38.88 ± 0.5**	351.8 ±17.2	35.86 ± 1.1

Data represented as mean ± SEM. *p<0.05, **p<0.01 (compared to UT), $^{\#}$p<0.05, $^{\#\#}$p<0.01 (compared to LD), unpaired t-test.

Table S8. Fiber size analysis of the gastrocnemius muscle of CAPN3KO mice from young age group at 20 weeks post-gene delivery and age-matched WT mice

	Untreated (n=8)		Low Dose (n=9)		High Dose (n=8)		WT (n=4)	
	**Number**	**Diameter (µm)**	**Number**	**Diameter (µm)**	**Number**	**Diameter (µm)**	**Number**	**Diameter (µm)**
**STO**	106.1 ± 6.8	25.17 ± 0.8	83.00 ± 5.7*	30.10 ± 0.9***	96.9 ± 8.2	25.20 ± 0.3$^{\#\#\#}$	131.3 ± 4.7*$^{\#\#\#\#\$}$	29.89 ± 0.2**$^{\$\$\$\$}$
**FTO**	53.5 ± 1.4	32.46 ± 0.8	75.2 ± 7.8*	35.99 ± 1.2*	84.5 ± 9.9*	32.00 ± 0.6$^{\#\#}$	38.8 ± 6.6*$^{\#\$}$	36.16 ± 0.8*$^{\$\$}$
**FTG**	196.3 ± 10.4	39.42 ± 1.2	153.9 ± 10.0*	44.70 ± 1.1**	161.4 ± 10.9*	42.50 ± 1.3	171.0 ± 5.4	42.40 ± 0.6
**All Fibers**	355.9 ± 15.0	34.12 ± 0.9	312.1 ± 12.4**	38.75 ± 1.0**	341.4 ± 12.5	35.29 ± 0.6$^{\#}$	341.0 ± 6.6	36.84 ± 0.3

Data represented as mean ± SEM. *p<0.05, **p<0.01 (compared to UT), $^{\#}$p<0.05, $^{\#\#}$p<0.01 (compared to LD), $^{\$}$p<0.05, $^{\$\$}$p<0.01 (compared to HD), unpaired t-test.

Table S9. Fiber type distribution (%) in the tibialis anterior muscle of CAPN3KO mice from old group and age-matched WT mice.

	UT	LD	HD	WT
**STO**	27.1 ± 1.4	30.2 ± 2.1	27.7± 2.4	28.1 ± 2.2
**FTO**	27.6 ± 1.3	28.2 ± 1.1	31.1 ± 1.5	$20.7 \pm 0.9^{**\#\#\#\$\$\$\$}$
**FTG**	45.4 ± 1.4	41.6 ± 2.5	41.2 ± 2.2	$51.1 \pm 2.0^{*\#\$\$}$

Data represented as mean ± SEM. *p<0.05, **p<0.01 (compared to UT), $^{\#}p<0.05$, $^{\#\#}p<0.01$ (compared to LD), $^{\$}p<0.05$, $^{\$\$}p<0.01$ (compared to HD), unpaired t-test.

Table S10. Fiber type distribution (%) in the gastrocnemius muscle of CAPN3KO mice from old group and age-matched WT mice

	UT	LD	HD	WT
**STO**	32.0 ± 2.1	33.5 ± 1.8	38.0 ± 1.6	31.6 ± 1.7
**FTO**	20.3 ± 1.1	17.8 ± 1.1	19.0 ± 1.1	$15.1 \pm 0.6^{**\$}$
**FTG**	47.7 ± 2.5	48.7 ± 2.0	43.1 ± 1.7	$53.2 \pm 1.6^{\$\$}$

Data represented as mean ± SEM. *p<0.05, **p<0.01 (compared to UT), $^{\#}p<0.05$, $^{\#\#}p<0.01$ (compared to LD), $^{\$}p<0.05$, $^{\$\$}p<0.01$ (compared to HD), unpaired t-test.

Table S11. Fiber type distribution (%) in the tibialis anterior muscle of CAPN3KO mice from young group and age-matched WT mice

	UT	LD	HD
**STO**	18.0 ± 2.1	16.4 ± 1.5	26.8 ± 1.2*$^{\#\#}$
**FTO**	20.8 ± 0.6	25.6 ± 1.4	24.8 ± 0.8*
**FTG**	61.2 ± 2.1	55.5 ± 3.1	48.4 ± 1.0**

Data represented as mean ± SEM. *p<0.05, **p<0.01 (compared to UT), $^{\#}$p<0.05, $^{\#\#}$p<0.01 (compared to LD), unpaired t-test.

Table S12. Fiber type distribution (%) in the gastrocnemius muscle of CAPN3KO mice from young group and age-matched WT mice

	UT	LD	HD	WT
**STO**	29.8 ± 1.3	26.5 ± 1.1	28.2 ± 1.8	38.5 ± 1.0**$^{\#\#\#\#\#\$\$}$
**FTO**	15.2 ± 0.5	24.1 ± 2.4**	24.5 ± 2.2**	11.4 ± 2.0$^{\$\$\#\#}$
**FTG**	55.1 ± 1.3	49.4 ± 2.8	47.7 ± 3.3	50.1 ± 1.0*

Data represented as mean ± SEM. *p<0.05, **p<0.01 (compared to UT), $^{\#}$p<0.05, $^{\#\#}$p<0.01 (compared to LD), $^{\$}$p<0.05, $^{\$\$}$p<0.01 (compared to HD), unpaired t-test.

Table S13. Fiber type distribution (%) in the quadriceps muscle of CAPN3KO mice from old group and age-matched WT mice

	UT	HD	WT
**STO**	19.6 ± 1.5	19.5 ± 3.1	21.4 ± 2.5
**FTO**	16.5 ± 1.2	19.4 ± 1.6	15.4 ± 1.6
**FTG**	64.0 ± 1.9	61.1 ± 2.8	63.2 ± 2.3

Data represented as mean ± SEM. Differences were not significant. Unpaired t-test.

Table S14. Fiber type distribution (%) in the triceps muscle of CAPN3KO mice from old group and age-matched WT mice.

	UT	LD	HD	WT
**STO**	26.2 ± 2.5	26.8 ± 1.8	24.5 ± 2.0	22.6 ± 1.2
**FTO**	24.5 ± 1.5	25.4 ± 1.3	24.2 ± 1.4	18.3 ± 0.4**$^{\#\#\$\$}$
**FTG**	49.3 ± 2.3	47.8 ± 2.7	51.3 ± 2.9	59.1 ± 1.0**$^{\#\#}$

Data represented as mean ± SEM. *p<0.05, **p<0.01 (compared to UT), $^{\#}$p<0.05, $^{\#\#}$p<0.01 (compared to LD), $^{\$}$p<0.05, $^{\$\$}$p<0.01 (compared to HD), unpaired t-test.

Table S15. Primers used in the study.

Target	Primer	Sequence (5' to 3')
AAVrh74	Forward	CGGAGAGCAACTGCATAAG
	Reverse	GGCTGATGATGGCTGAATAG
CAPN3	Forward	CTACGAGGTTCCCAAAGAGATG
	Reverse	CCCGCATGTTGATGTAGGT
PGC-1α	Forward	AATCAGACCTGACACAACGC
	Reverse	GCATTCCTCAATTTCACCAA

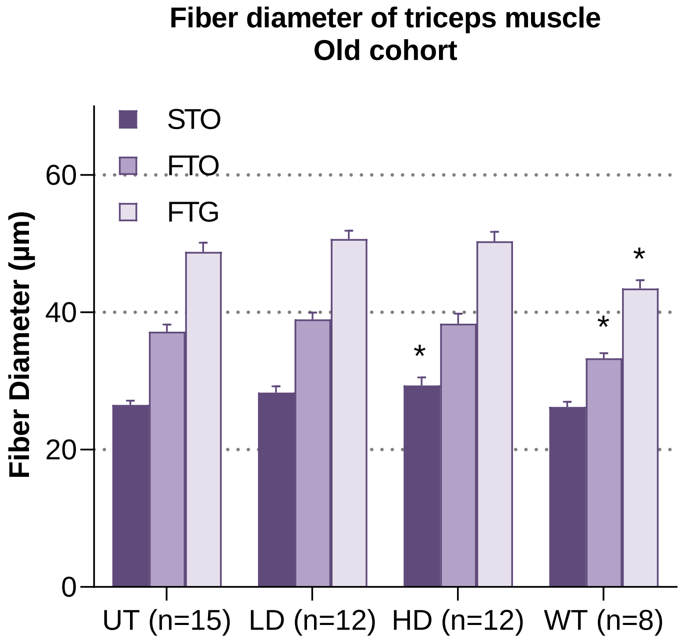

Figure S1. Fiber size analysis of the triceps muscle from old CAPN3KO mice at 20 weeks post-gene delivery and age-matched WT mice

Fiber size measurements, done on images derived from three zones of the triceps muscle showed significant increase in STO fiber in HD cohorts compared to UT CAPN3KO group. Each bar represents mean ± SEM. *p<0.05, (compared to UT), unpaired t-test.

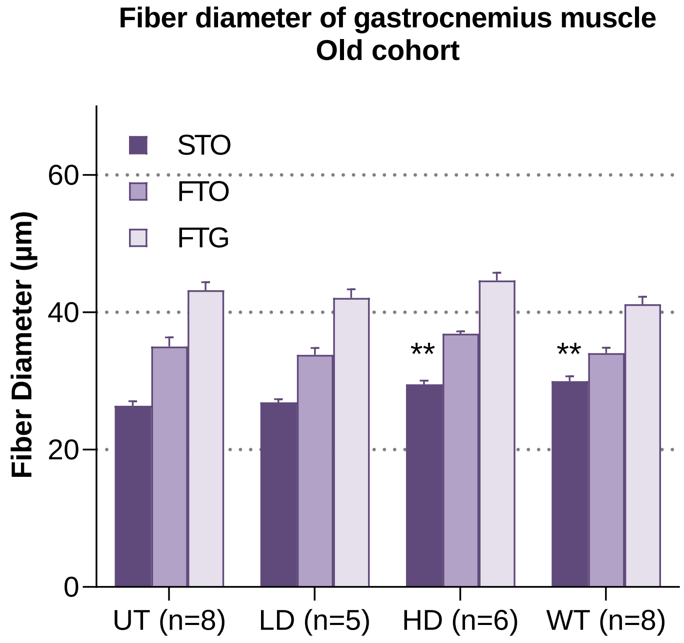

Figure S2. Fiber size analysis of the gastrocnemius muscle from old CAPN3KO mice at 20 weeks post-gene delivery and age-matched WT mice

Fiber size measurements, done on images derived from three zones of the gastrocnemius muscle showed significant increase in STO fibers in HD cohorts compared to UT CAPN3KO group. Each bar represents mean ± SEM. **p<0.01, (compared to UT), unpaired t-test.

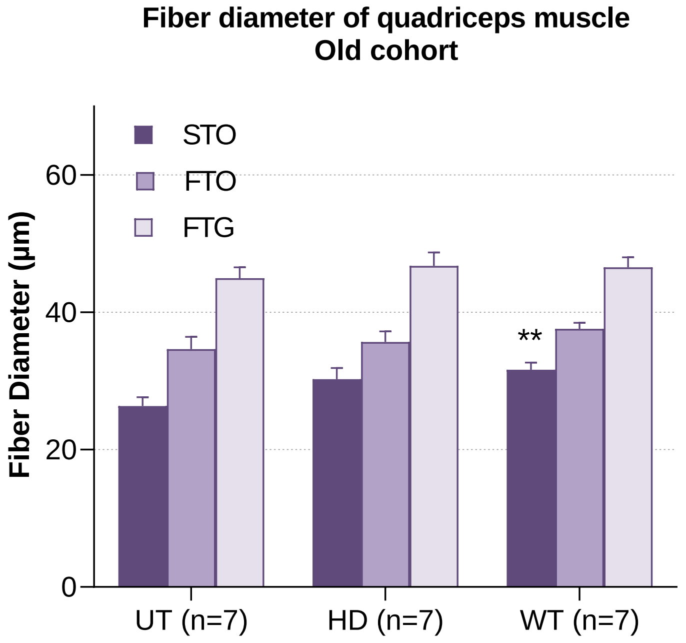

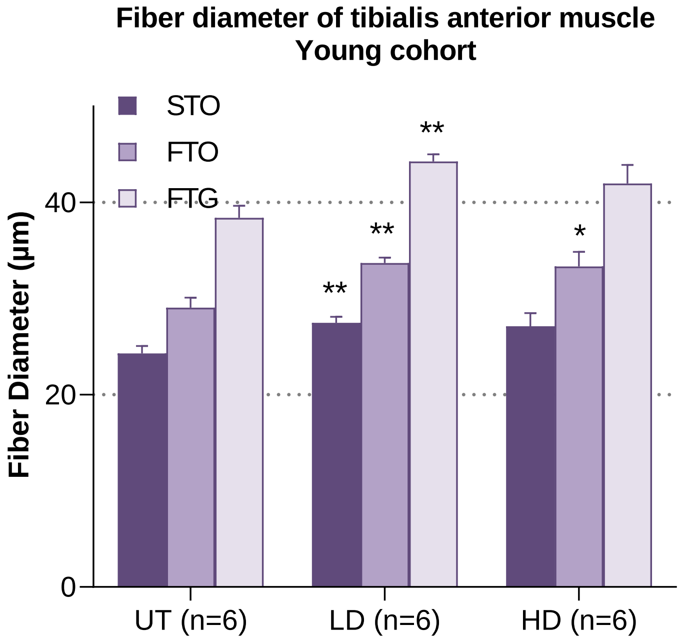

Figure S3. Fiber size analysis of the quadriceps muscle from old CAPN3KO mice at 20 weeks post-gene delivery and age-matched WT mice

Fiber size measurements, done on images derived from three zones of the quadriceps muscle showed an increasing trend for all fiber types (STO, FTO and FTG) in LD cohort compared to UT CAPN3KO group. Each bar represents mean ± SEM. **$p<0.01$ (compared to UT), unpaired t-test.

Figure S4. Fiber size analysis of the tibialis anterior muscle from young CAPN3KO mice at 20 weeks post-gene delivery and age-matched WT mice

Fiber size measurements, done on images derived from three zones of the triceps muscle showed significant increases for all fiber types (STO, FTO and FTG) in LD cohort and increase in FTO fibers in HD cohort compared to UT CAPN3KO group. Each bar represents mean ± SEM. *$p<0.05$, **$p<0.01$ (compared to UT), unpaired t-test.

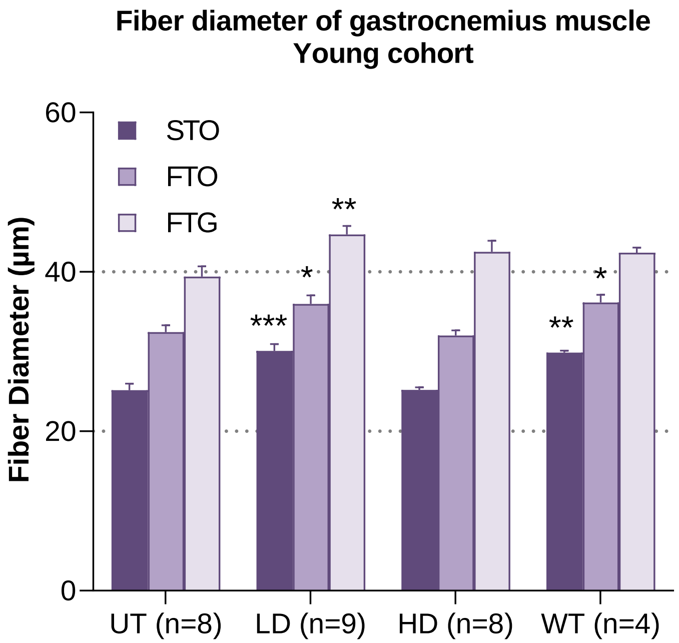

Figure S5. Fiber size analysis of the gastrocnemius muscle from young CAPN3KO mice at 20 weeks post-gene delivery and age-matched WT mice

Fiber size measurements, done on images derived from three zones of the triceps muscle showed significant increases for all fiber types (STO, FTO and FTG) in LD cohort compared to UT CAPN3KO group. Each bar represents mean ± SEM. *p<0.05, **p<0.01, ***p<0.001 (compared to UT), unpaired t-test.

		OLD				YOUNG			
		TA (UT:15, LD:12, HD:12)		Gas (UT:8, LD:4, HD:6)		TA (UT:6, LD:6, HD:6)		Gas (UT:8, LD:4, HD:6)	
		LD	HD	LD	HD	LD	HD	LD	HD
STO	Size	↑*	↑*	≈↑	↑*	↑*	↑	↑*	≈↑
		↑*	↑*	≈↑	↑*	↑	↑*	↑*	↑
		↑*	↑*	≈↓	↑	↑	↑	↑	↓
	Percent	↑	≈↑	↑	↑	↓	↑*	↓	↑
		↑	↑	↑	↑*	↓	↑	↓	↓
		↓	↓	↓	↓	↓	↑*	↓*	↓
FTO	Size	↑*	↑*	↓	↑	↑*	↑*	↑*	≈↓
		↑*	↑	≈↓	↑*	↑	↑	↑	≈↑
		↑	↑*	↓	≈↓	↑	↑	↑	↓
	Percent	↑	↑	↓	↓	↑	↑*	↑*	↑*
		↑	↑	↓	↓	↑	↑	↑	↑*
		↓	↑	↓	↓	↑	↑	↑*	↑*
FTG	Size	↑*	↑*	≈↓	↑	↑*	↑	↑*	↑
		↑*	↑*	≈↑	↑	↑*	↑	↑*	↑*
		↑	↑*	↓	↓	↑	↑	↑	≈↑
	Percent	↓	↓	↑	↓	↓	↓*	↓	↓
		↓*	↓*	↓	↓	↓	↓	↓	↓
		↑	≈↓	↑	↑*	↓	↓*	↓	↓

Figure S6. Muscle Fiber Type Remodeling in CAPN3KO Mice with AAV.CAPN3 Gene Therapy

Compared to untreated (UT) cohort, direction of changes observed in the mean fiber size and percent distribution of each fiber type in tibialis anterior (TA) and gastrocnemius (Gas) muscles from the treated CAPN3KO mice of both age groups are depicted with arrows. Black arrows indicate the direction of change for all mice analyzed within the corresponding group, red arrows represent female mice and blue arrows represent male mice. Asterisk indicates that the change observed is significantly different from untreated mice.

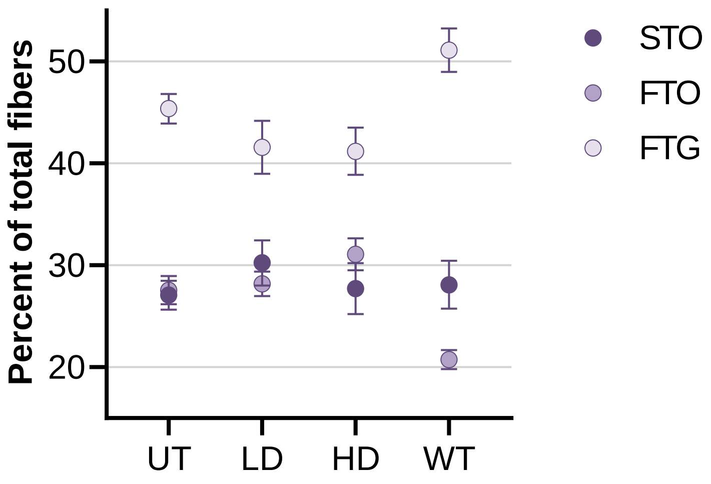

Figure S7. Fiber type distribution (%) in the tibialis anterior muscle of CAPN3KO mice from old group and age-matched WT mice

Graph shows the percent changes in the fiber type (STO, FTO and FTG) distribution in the tibialis anterior muscle from UT, LD and HD treatment cohorts and age-matched WT mice. Data are represented as mean ± SEM. For statistics, please see Table S9.

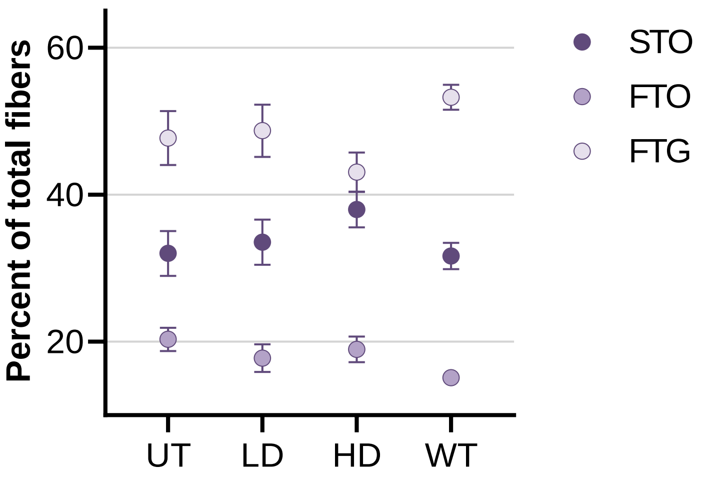

Figure S8. Fiber type distribution (%) in the gastrocnemius muscle of CAPN3KO mice from old group and age-matched WT mice

Graph shows the percent changes in the fiber type (STO, FTO and FTG) distribution in the gastrocnemius muscle from UT, LD and HD treatment cohorts and age-matched WT mice. Data are represented as mean ± SEM. For statistics, please see Table S10.

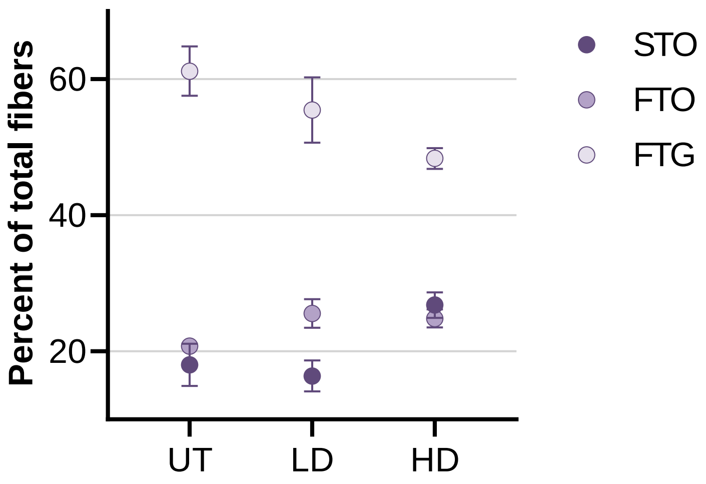

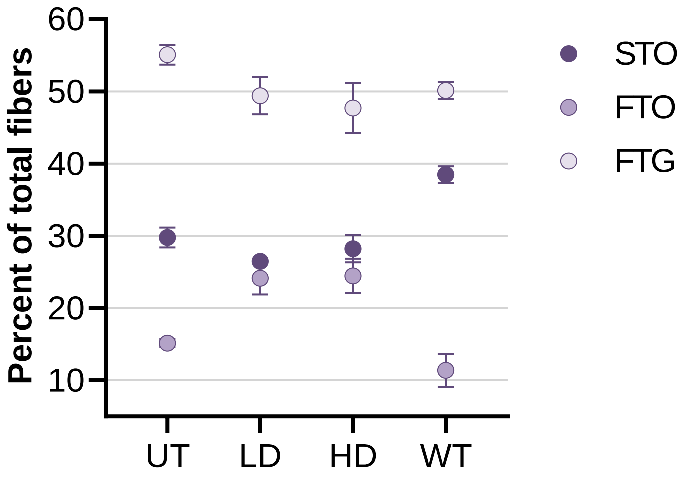

Figure S9. Fiber type distribution (%) in the gastrocnemius muscle of CAPN3KO mice from old group and age-matched WT mice
Graph shows the percent changes in the fiber type (STO, FTO and FTG) distribution in the gastrocnemius muscle from UT, LD and HD treatment cohorts and age-matched WT mice. Data are represented as mean ± SEM. For statistics, please see Table S11.

Figure S10. Fiber type distribution (%) in the gastrocnemius muscle of CAPN3KO mice from old group and age-matched WT mice
Graph shows the percent changes in the fiber type (STO, FTO and FTG) distribution in the gastrocnemius muscle from UT, LD and HD treatment cohorts and age-matched WT mice. Data are represented as mean ± SEM. For statistics, please see Table S12.

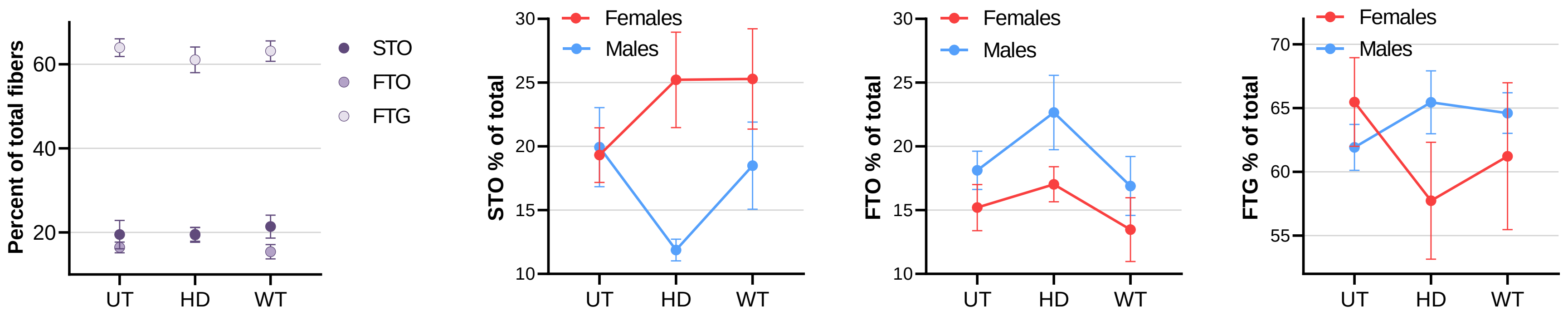

Figure S11. Fiber type distribution (%) in the quadriceps muscle of CAPN3KO mice from old group and age-matched WT mice
Graphs represent the percent changes in the fiber type (STO, FTO and FTG) distribution in the quadriceps muscle from UT cohort, HD treatment cohort and age-matched WT mice, shown as sexes combined (A) and separated (B-D). Data are represented as mean ± SEM, unpaired t-test.

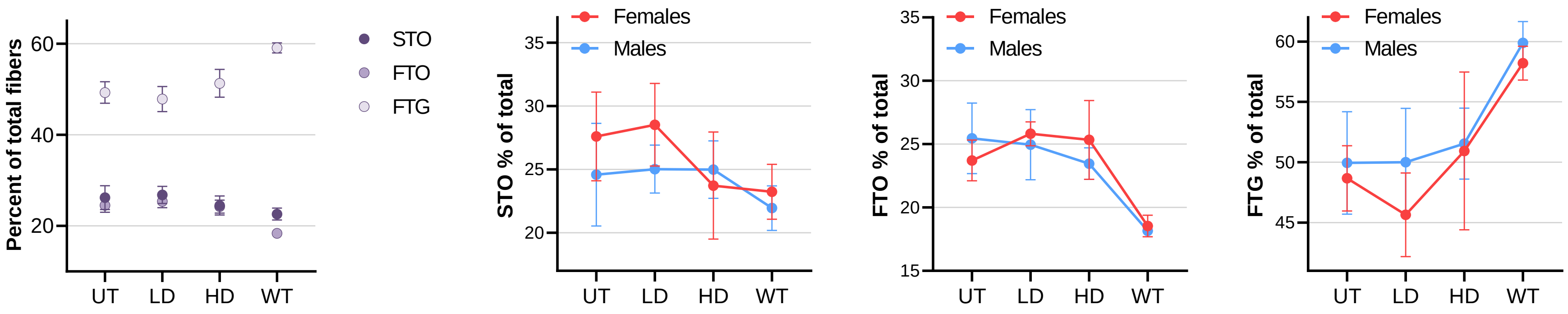

Figure S12. Fiber type distribution (%) in the triceps muscle of CAPN3KO mice from old group and age-matched WT mice
Graphs represent the percent changes in the fiber type (STO, FTO and FTG) distribution in the triceps muscle from UT cohort, HD treatment cohort and age-matched WT mice, shown as sexes combined (A) and separated (B-D). Data are represented as mean ± SEM, *p<0.05, **p<0.01, ***p<0.001 unpaired t-test. For statistics of panel A, please see Table S14.

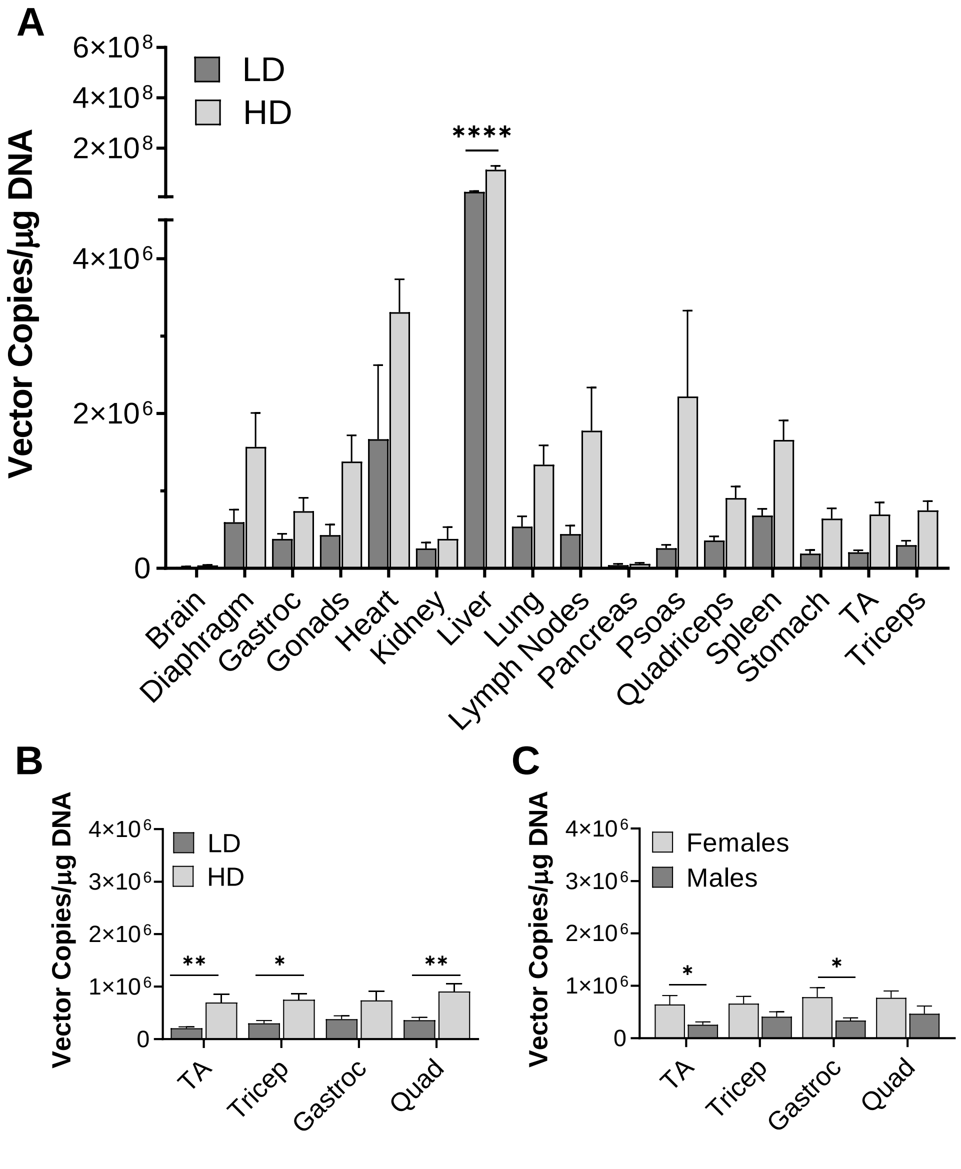

Figure S13. AAVrh74.tMCK.hCAPN3 Vector Biodistribution 20 Weeks Post-injection
(A-C) Vector copy number which corresponds to viral genomes/diploid genome (vg/dg or vector copies per µg of genomic DNA), was quantified in various tissues including four different muscle groups from lower and upper limbs from CAPN3KO mice of LD and HD cohorts of old age group (A) Vector genome distribution was variable in organs and muscles in a dose dependent manner. The highest vector genome copy number was present in the liver from both treatment cohorts. (B) The HD treatment resulted in a 59.6% increase in skeletal muscle vg levels, $p < 0.0001$, compared with LD. (C) Gender difference in the AAV biodistribution in CAPN3KO is shown. Each bar represents the mean ± SEM of 9 to 12 samples. *$p<0.05$, **$p<0.01$, ****$p<0.0001$, two-way ANOVA, Tukey's multiple comparisons test.
